# Supplementary material for: SETBP1 variants outside the degron disrupt DNA-binding, transcription and neuronal differentiation capacity to cause a heterogeneous neurodevelopmental disorder
Source: Nat Commun. 2025 Oct 10;16:9021. doi: 10.1038/s41467-025-64074-x (PMC12514306; doi:10.1038/s41467-025-64074-x)

Supplementary Information

SETBP1 variants outside the degron disrupt DNA-binding and transcription to cause a heterogeneous neurodevelopmental disorder

Maggie MK Wong,[1,*] Rosalie A Kampen,[1] Ruth O Braden,[2,3] Gökberk Alagöz,[1] Michael S Hildebrand,[2,4] Alexander JM Dingemans,[5] Jean Corbally,[1] Joery den Hoed,[1] Ezequiel Mendoza,[6] Willemijn JJ Claassen,[1] Christopher Barnett,[7] Meghan Barnett,[7] Alfredo Brusco,[8,9] Diana Carli,[10,11] Bert BA de Vries,[5] Frances Elmslie,[12] Giovanni Battista Ferrero,[13] Nadieh A Jansen,[5] Ingrid MBH van de Laar,[14] Alice Moroni,[8] David Mowat,[15,16] Lucinda Murray,[17] Francesca Novara,[18] Angela Peron,[19,20,21] Ingrid E Scheffer,[2] Fabio Sirchia,[22] Samantha J Turner,[2,3] Aglaia Vignoli,[23] Arianna Vino,[1] Sacha Weber,[24] Wendy K Chung,[25,26] Marion Gerard,[24] Vanesa López-González,[27] Elizabeth Palmer,[16,17] Angela T Morgan,[3,28] Bregje W van Bon,[5] Simon E Fisher[1,29,*]

*Correspondence: maggie.wong@mpi.nl and simon.fisher@mpi.nl

This file includes:

Supplementary Fig 1 – 14

Supplementary references

Source data for Supplementary Fig 3D

Supplementary Figure 1

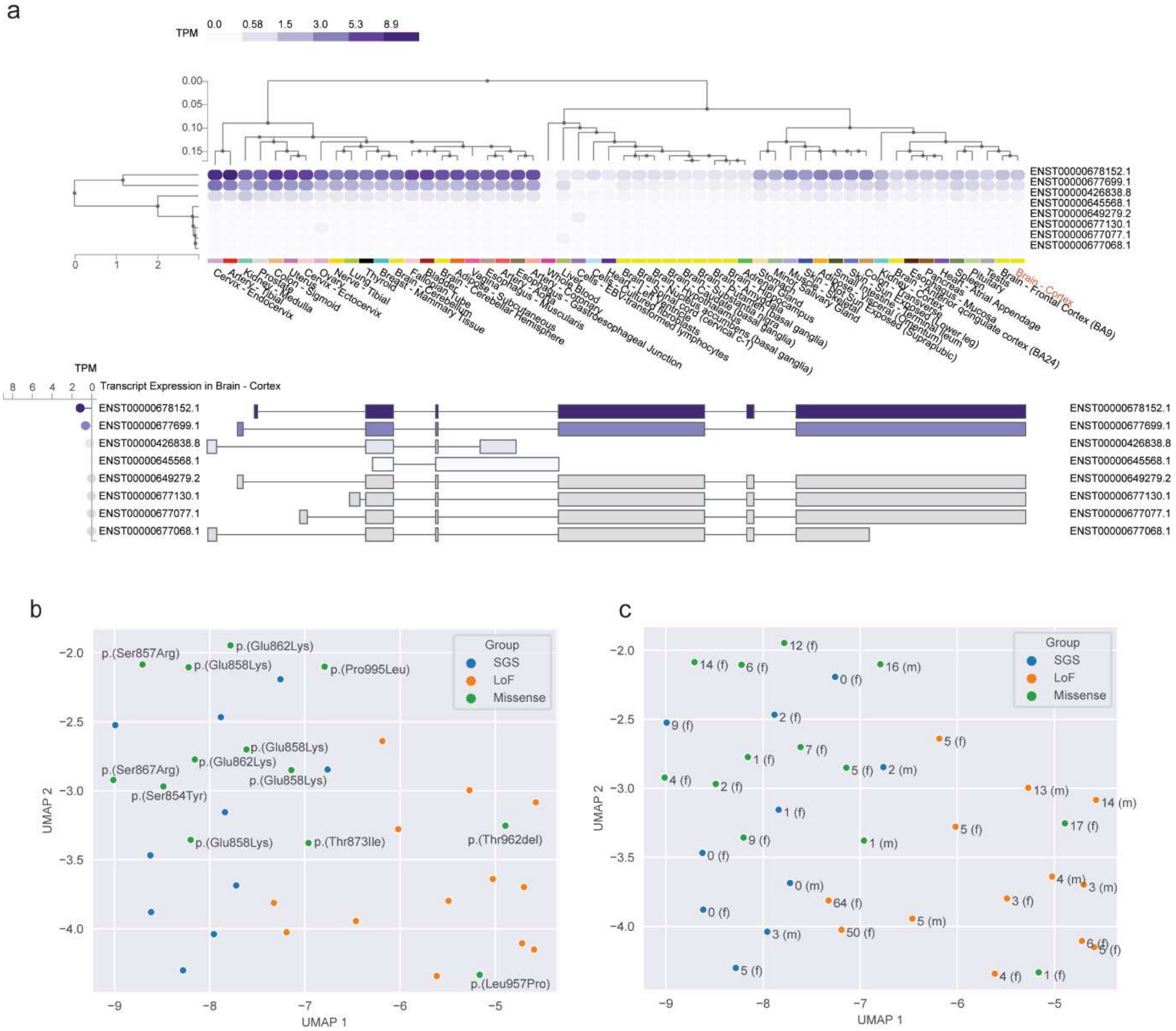

Supplementary Figure 1: *SETBP1* isoform expression and PhenoScore prediction of *SETBP1* missense variants outside the degron.

a) Isoform expression of *SETBP1* in adult human tissues from the GTEx transcript browser. Expression in the cortex is highlighted.

b) UMAP of PhenoScore prediction of individuals with *SETBP1* missense variants outside the degron labelled with variants. SGS and LoF variants cluster separately. Some missense variants outside the degron are more similar to LoF variants. Some are more similar to SGS but those located at the top cluster with neither SGS nor LoF variants.

c) UMAP of PhenoScore prediction of individuals with *SETBP1* missense variants outside the degron labelled with age and biological sex. No age or biological sex-related bias was detected.

Source data are provided as a Source Data file.

Supplementary Figure 2

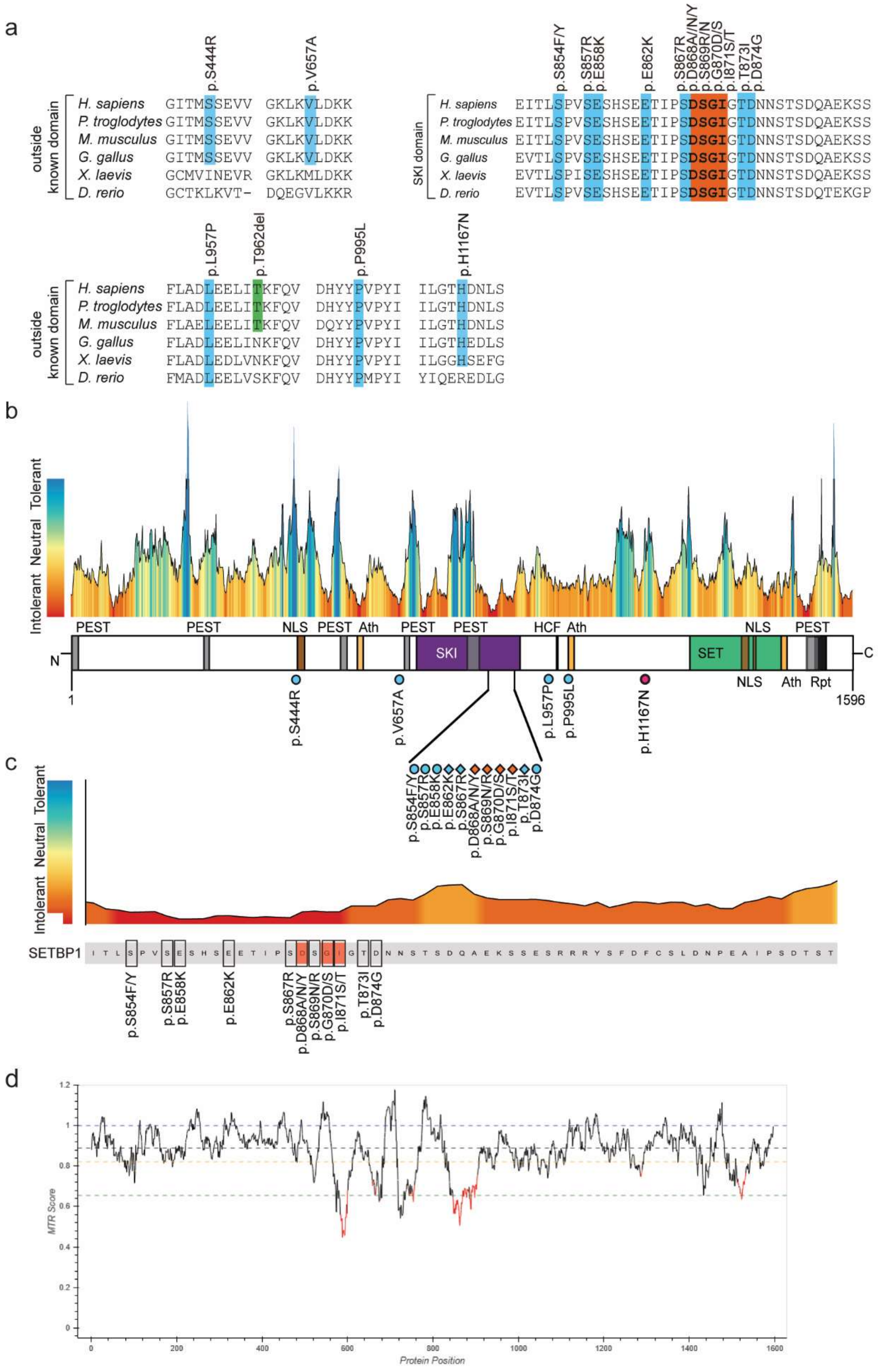

Supplementary Figure 2: *In silico* analysis of *SETBP1* missense variants.

a) Sequence alignment of the region containing part of the SETBP1 amino acid sequence in human (Uniprot: Q9Y6X0), chimpanzee (H2QEG8), mouse (Q9Z180), chicken (A0A1D5PT15), African clawed frog (F6TBV9), and zebrafish (B0R147). The canonical degron is highlighted in bold. Residues are highlighted for which germline missense variants are seen, located within the degron (orange), outside the degron (blue), or deleted by an in-frame deletion (green).

b) Overview of the SETBP1 protein (transcript NM_015559.2, ENST00000282030.5) intolerance landscape visualized via the MetaDome web server version 1.0.1. The tolerance landscape is computed based on single-nucleotide variants present in the gnomAD database. It is calculated as a missense over synonymous ratio in a sliding window of 21 residues over the entire *SETBP1* protein. The green and blue peaks correspond to regions that are more tolerant to missense variation, and the red valleys indicate intolerant regions. The locations of the variants in our cohort are displayed within the tolerance landscape of SETBP1. Most of these variants are predicted to be intolerant to highly intolerant to missense variation. Previously reported SGS variants located within the degron are also indicated.

c) Detailed overview of the intolerance landscape of the SETBP1 SKI domain where missense variants cluster. The canonical degron is highlighted.

d) Predicted regional intolerance of SETBP1 missense variants using Missense Tolerance Ratio (MTR)[1] v2, a measure of regional intolerance to missense variation calculated using variation from 240 000 exome and genome sequences (overlay with all populations). Horizontal lines show gene-specific MTR percentiles 5th, 25th, 50th, and neutrality (MTR = 1.0). MTR is calculated using the WES component of gnomAD v2.0. Less tolerated regions are highlighted in red.

Source data are provided as a Source Data file.

Supplementary Figure 3

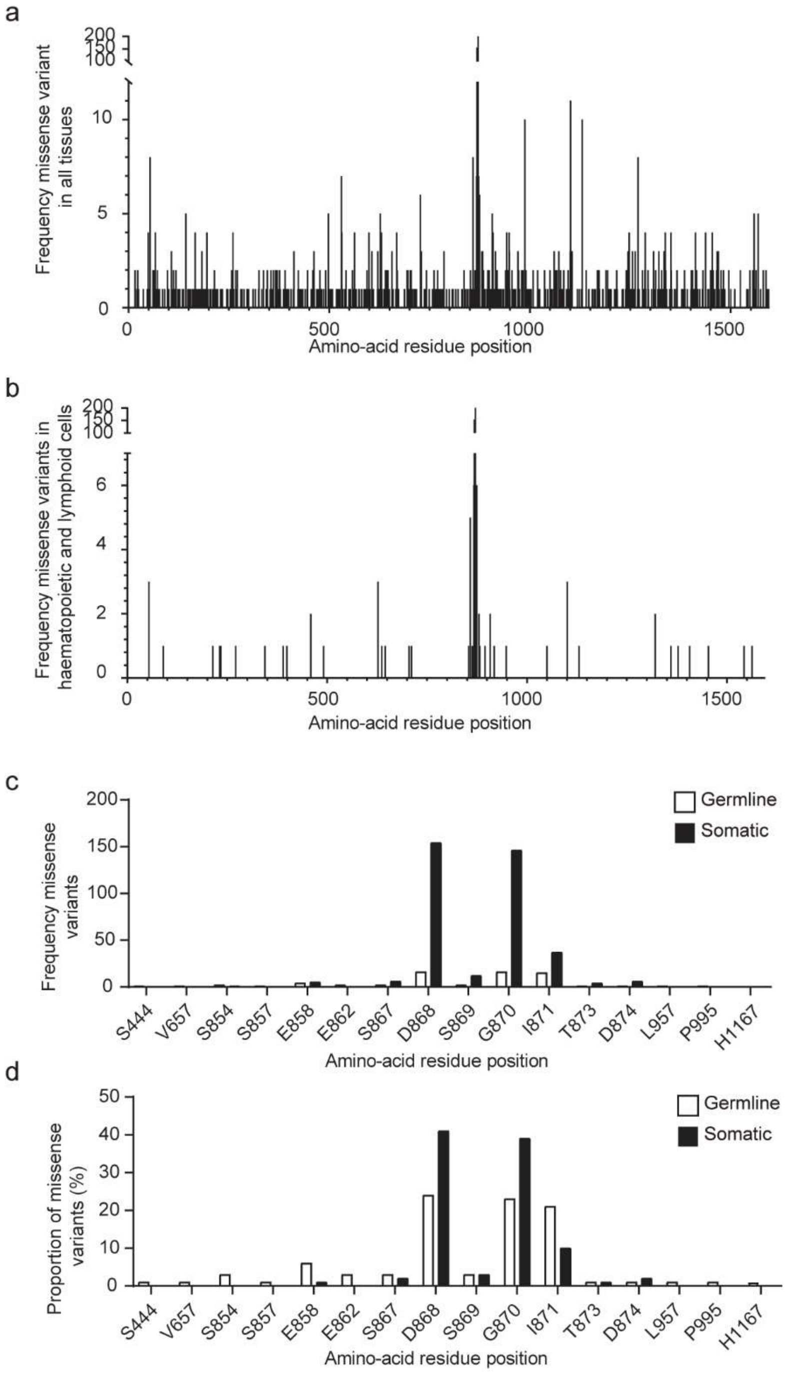

Supplementary Figure 3: Frequency of somatic and germline *SETBP1* variants.

a) Frequency of all *SETBP1* missense variants in all tissues.

b) Frequency of all *SETBP1* missense variants in haematopoietic and lymphoid cells.

c) Frequency of germline vs somatic missense variants included in this study.

d) Proportion of germline vs somatic missense variants included in this study.

Source data are provided as a Source Data file.

Supplementary Figure 4

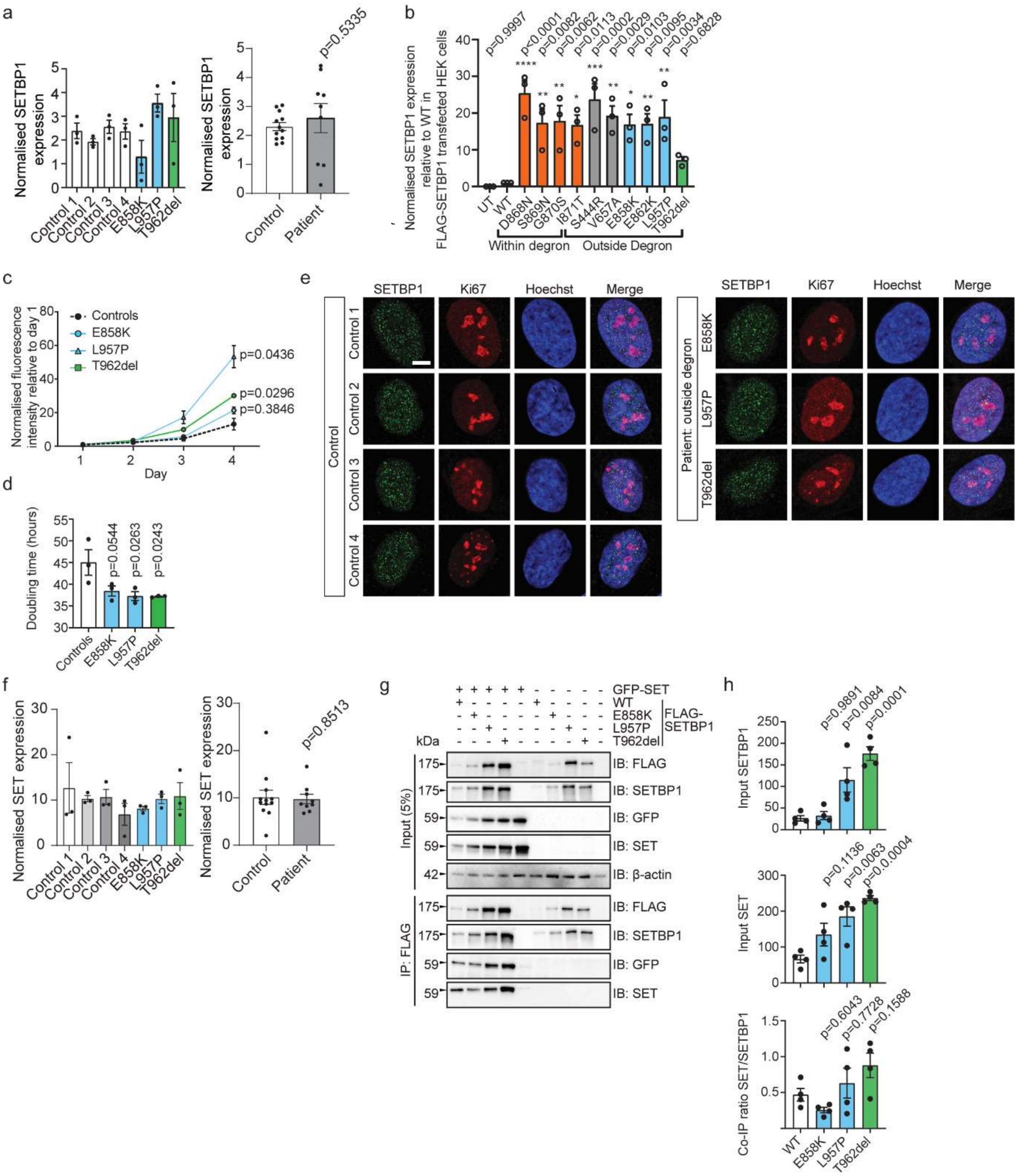

Supplementary Figure 4: Expression of *SETBP1* and *SET* and increased proliferation of fibroblasts derived from patients carrying *SETBP1* variants outside the canonical degron

a) Normalised *SETBP1* transcript expression (bottom) in control and patient HDFs. Bars represent the mean ±SEM of three independent experiments (left: not significant, versus controls, one-way ANOVA and a *post-hoc* Dunnett's test; right: versus control, student's t-test).

b) Normalised expression of *SETBP1* relative to WT in HEK cells transiently expressing a FLAG-SETBP1 plasmid. Bars represent the mean ±SEM of three independent experiments (versus WT, one-way ANOVA and a *post-hoc* Dunnett's test).

c) CyQuant cell proliferation assay of fibroblasts from healthy individuals (controls) and patients carrying a *SETBP1* variant outside the degron. Nuclei of fibroblasts were stained with a GFP fluorescence dye. Fluorescence activity was measured daily for four days. Values are expressed relative to 1 day after seeding (day 1) and represent the mean ± SEM of three independent experiments, each performed in triplicate (versus controls; two-way ANOVA and a *post-hoc* Tukey's test).

d) Cell doubling time of fibroblasts. Values represent the mean ± SEM of three independent experiments (versus healthy controls; one-way ANOVA and a *post-hoc* Dunnett's test).

e) Confocal microscopy images of immunostained SETBP1 (green) and proliferation marker Ki67 (red) in control and patient fibroblasts. Nuclei were stained with Hoechst 33342 (blue). Merged images are shown. Ki67 shows a characteristic blob-like pattern in the nucleus in S phrase in all variants. Results are representative of three independent experiments. Scale bar = 5μm.

f) Normalised *SET* transcript expression (bottom) in control and patient HDFs. Bars represent the mean ±SEM of three independent experiments (left: not significant, versus controls, one-way ANOVA and a *post-hoc* Dunnett's test; right: versus control, student's t-test).

g) Co-immunoprecipitation (Co-IP) was performed in whole cell lysates co-expressing FLAG-*SETBP1* and GFP-SET. Wild type FLAG-*SETBP1* and variants were co-immunoprecipitated using FLAG-conjugated magnetic agarose. Immunoblots were probed with an anti-FLAG, anti-*SETBP1*, anti-GFP, or anti-SET antibody. β-actin was used as the loading control in the input fraction.

h) Quantification of *SETBP1* (top) and SET (middle) levels in the input fraction. Quantification of SET co-immunoprecipitated with *SETBP1* in the IP fraction was plotted (bottom). Values are expressed as the mean ± SEM of four independent experiments (versus WT; one-way ANOVA and a *post-hoc* Dunnett's test).

Source data are provided as a Source Data file.

Supplementary Figure 5

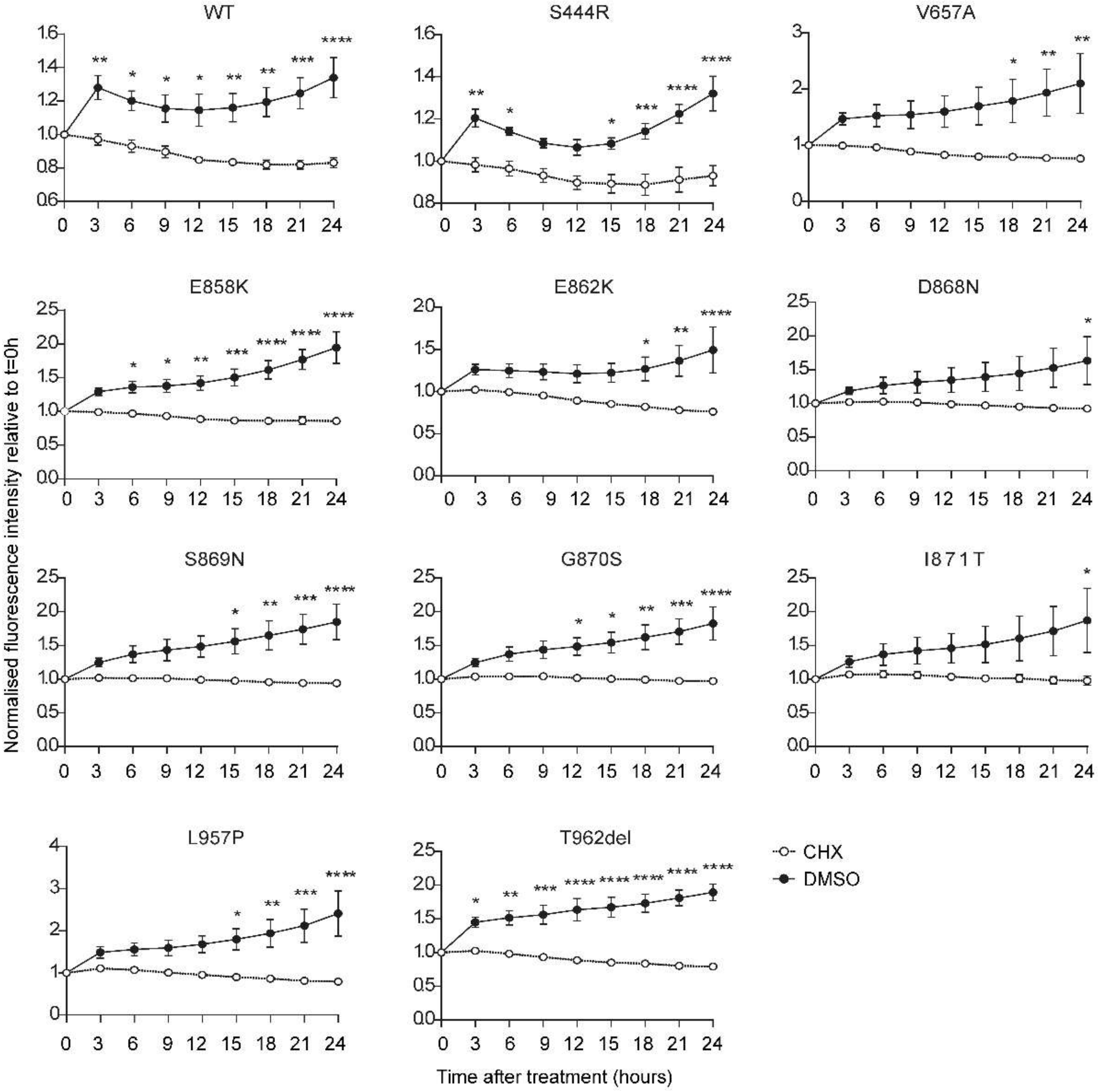

Supplementary Figure 5: Relative expression of *SETBP1* variants as YFP-fusion protein in HEK293T/17 cells treated with 50µg/mL cycloheximide (CHX) or DMSO vehicle control. Supplementary Fig for Fig 3b (top).

Normalized fluorescence intensity of YFP-*SETBP1* in living HEK293T cells treated with 50µg/mL cycloheximide (CHX) or equal volume of DMSO as vehicle control. Fluorescence intensity was measured for 24 hours with three-hour intervals and normalised to the transfection control mCherry. Values are expressed relative to t = 0 hour and represent the mean ± SEM of three independent experiments, each performed in triplicate (**p*<0.05, ***p*<0.01, ****p*<0.001, *****p*<0.0001 CHX versus DMSO; repeated measure two-way ANOVA and a *post-hoc* Sidak's test). Source data are provided as a Source Data file.

Supplementary Figure 6

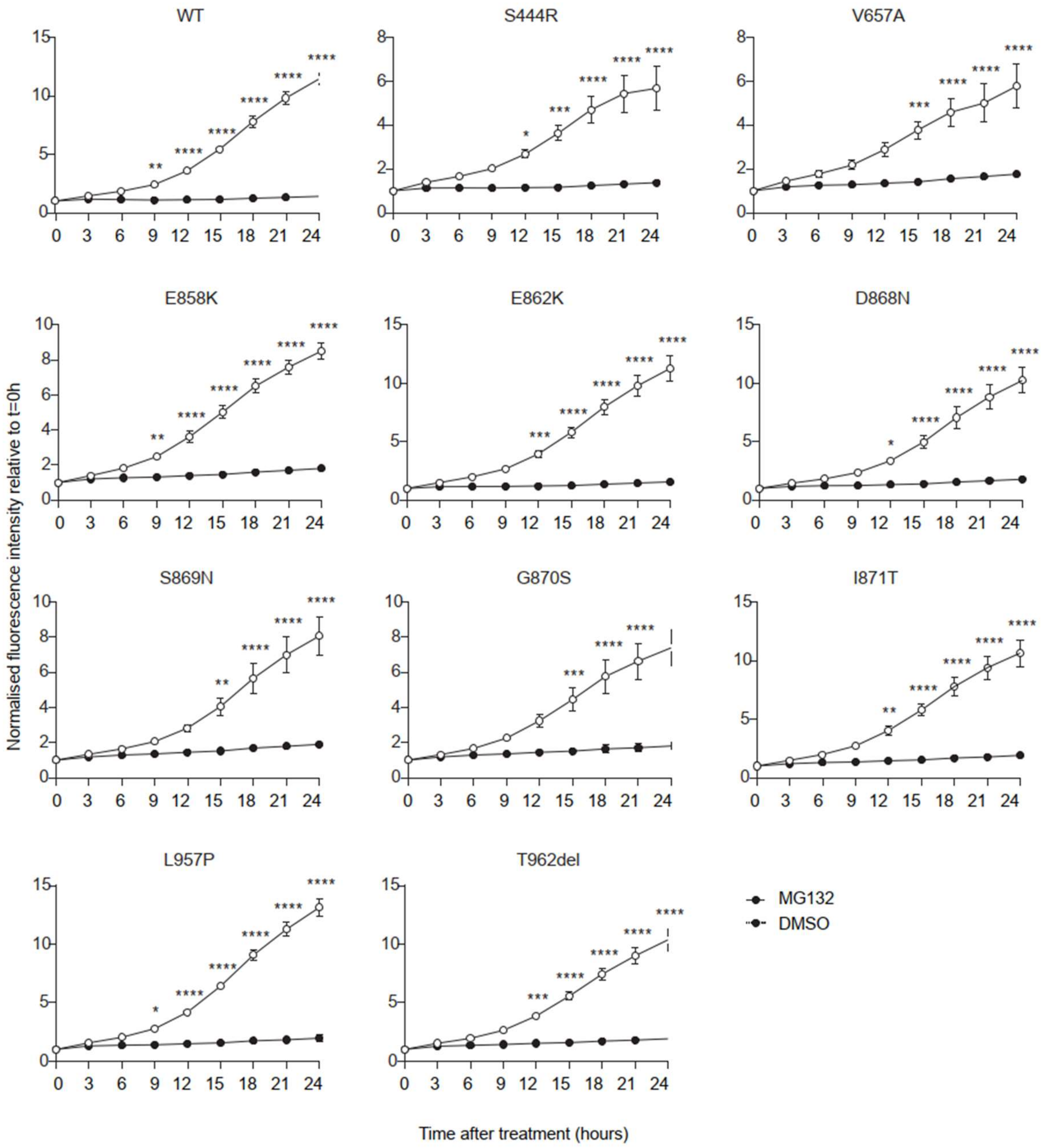

Supplementary Figure 6: Relative expression of *SETBP1* variants as YFP-fusion protein in HEK293T/17 cells treated with 5μg/mL MG132 or DMSO vehicle control. Supplementary Fig for Fig 3b (middle).

Normalized fluorescence intensity of YFP-*SETBP1* in living HEK293T cells treated with 5μg/mL MG132 or equal volume of DMSO as vehicle control. Fluorescence intensity was measured for 24 hours with three-hour intervals and normalised to transfection control mCherry. Values are expressed relative to t = 0 hour and represent the mean ± SEM of three independent experiments, each performed in triplicate (**p*<0.05, ***p*<0.01, ****p*<0.001, *****p*<0.0001 MG132 versus DMSO; repeated measure two-way ANOVA and a *post-hoc* Sidak's test). Source data are provided as a Source Data file.

Supplementary Figure 7

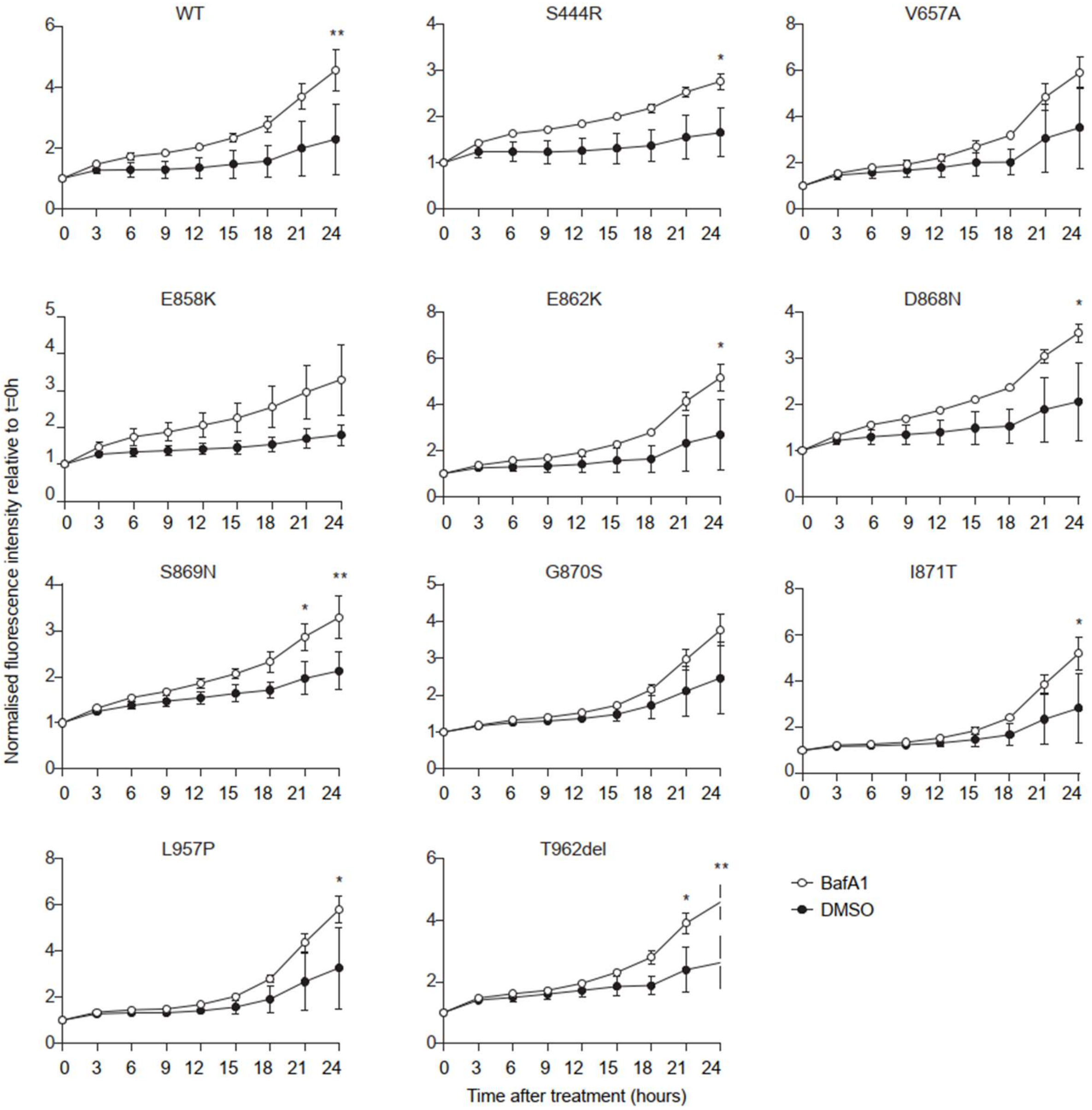

Supplementary Figure 7: Relative expression of *SETBP1* variants as YFP-fusion protein in HEK293T/17 cells treated with 100nM Bafilomycin (BafA1) or DMSO vehicle control. Supplementary Fig for Fig 3b (right).

Normalized fluorescence intensity of YFP-*SETBP1* in living HEK293T cells treated with 100nM BafA1 or equal volume of DMSO as vehicle control. Fluorescence intensity was measured for 24 hours with three-hour intervals and normalised to transfection control mCherry. Values are expressed relative to t = 0 hour and represent the mean ± SEM of three independent experiments, each performed in triplicate (**p*<0.05, ***p*<0.01, BafA1 versus DMSO; repeated measure two-way ANOVA and a *post-hoc* Sidak's test). Source data are provided as a Source Data file.

Supplementary Figure 8

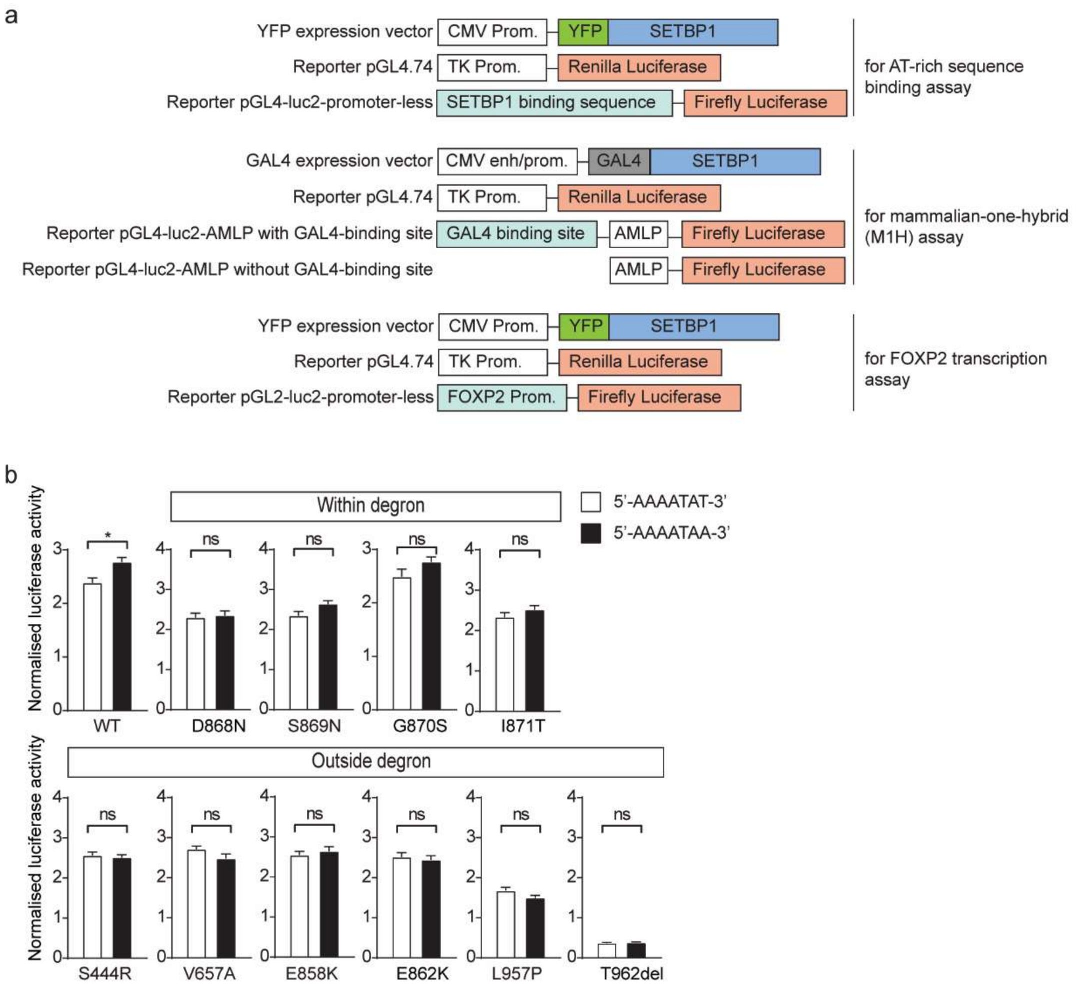

Supplementary Figure 8: *SETBP1* affinity to two AT-rich DNA consensus sequences.
a) Expression constructs used in the luciferase reporter assays: a YFP-fused *SETBP1* construct under a CMV promoter; a control construct with Renilla luciferase under control of a TK promoter for normalisation; and a promoter-less Firefly luciferase reporter construct carrying six repeats of 5'-AAAATAA-3' or 5'-AAAATAT-3' consensus *SETBP1* binding sequence previously reported in Piazza et al., 2018[2] (top panel). In the mammalian-one-hybrid (M1H) assay, a GAL4-fused *SETBP1* construct with a CMV enhancer and promoter; a control construct with Renilla luciferase under control of a TK promoter; a Firefly luciferase reporter construct with or without a GAL4-binding site and an adenovirus major late promoter (AMLP) (middle panel). Expression constructs used in the luciferase reporter assays: a YFP-fused *SETBP1* construct under a CMV promoter; a control construct with Renilla luciferase under control of a TK promoter for normalisation; and a promoter-less Firefly luciferase

reporter construct carrying FOXP2 promoters (TSS1 or TSS2) previously reported in Becker et al., 2018[3] (bottom panel).

b) Results of luciferase assays with pYFP constructs containing WT or *SETBP1* variants, and the Firefly luciferase reporter constructs carrying six repeats of 5'-AAAATAA-3' or 5'-AAAATAT-3' consensus *SETBP1* binding sequence previously reported in Piazza et al., 2018[2]. Values are expressed relative to the control condition that used a pCMV-YFP construct without *SETBP1* and represent the mean ± SEM of three independent experiments, each performed in triplicate (*$p<0.05$, student's t-test). Source data are provided as a Source Data file.

Supplementary Figure 9

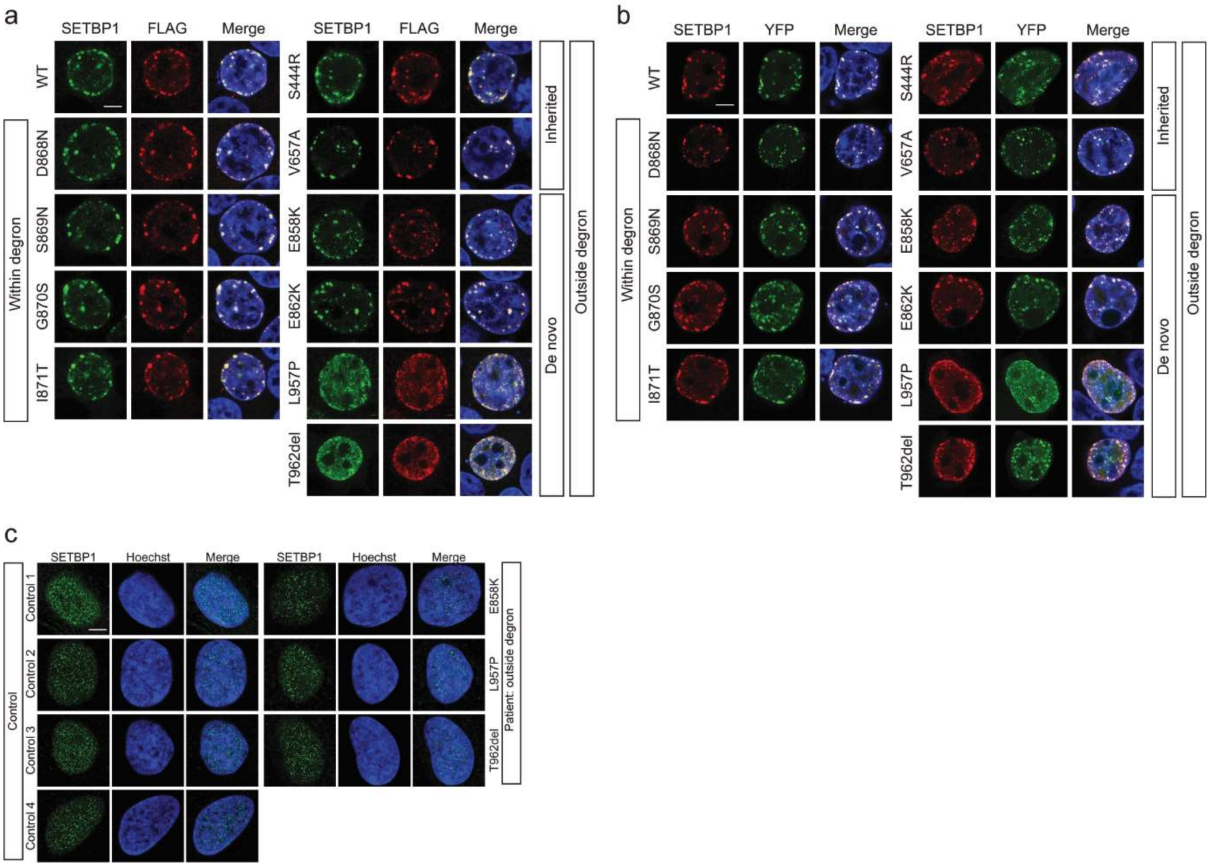

Supplementary Figure 9: *SETBP1* expression and subcellular localization in transiently transfected HEK293T/17 cells and human dermal fibroblasts.

a) Confocal microscopy images of *SETBP1* (green) and FLAG (red) localization of WT FLAG-*SETBP1* and variants. Results are representative of three independent experiments. Scale bar = 5µm.

b) Direct fluorescence imaging of cells expressing YFP-tagged variants of the *SETBP1* protein using confocal microscopy. Wildtype and all variants show a speckle-like pattern in the nucleus. Nuclei are stained with Hoechst 33342 (blue). Scale bars = 5µm.

c) Confocal microscopy images of immunostaining of *SETBP1* (green). Nuclei were stained with Hoechst 33342 (blue). Wildtype and all variants show a speckle-like pattern in the nucleus. Results are representative of three independent experiments. Scale bar = 5µm.

Supplementary Figure 10

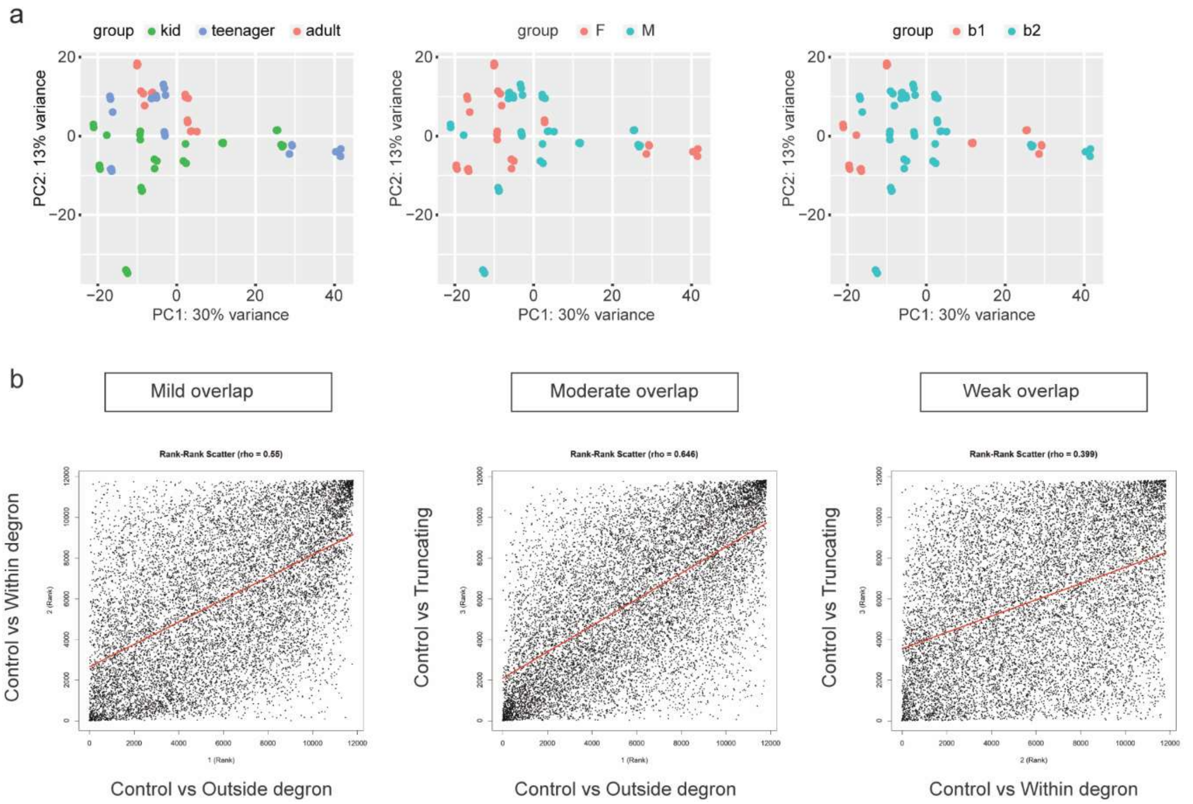

Supplementary Figure 10: Principal component analysis plots, Venn diagrams and RRHO plots of differentially expressed genes in fibroblasts (for Fig 5).

a) Principal component analysis (PCA) plots of variance distribution of fibroblast lines included in RNA-seq experiments. Three technical replicates were included for each line. Principal component (PC) 1 and PC2 account for 30% and 13% of total variance respectively . Samples were coloured by age group at sampling (left), biological sex (middle) and batch of sequencing (right). A list of cell lines included can be found in Supplementary Data 6. Kid = age 0-12; teenager = age 13-20; adult = age >20. F = female; M = male; b1 = batch 1; b2 = batch 2. b) Scatter plots of RRHO analyses. *Rho* indicates Spearman rank correlation coefficient between two comparisons.

Source data are provided as a Source Data file.

Supplementary Figure 11

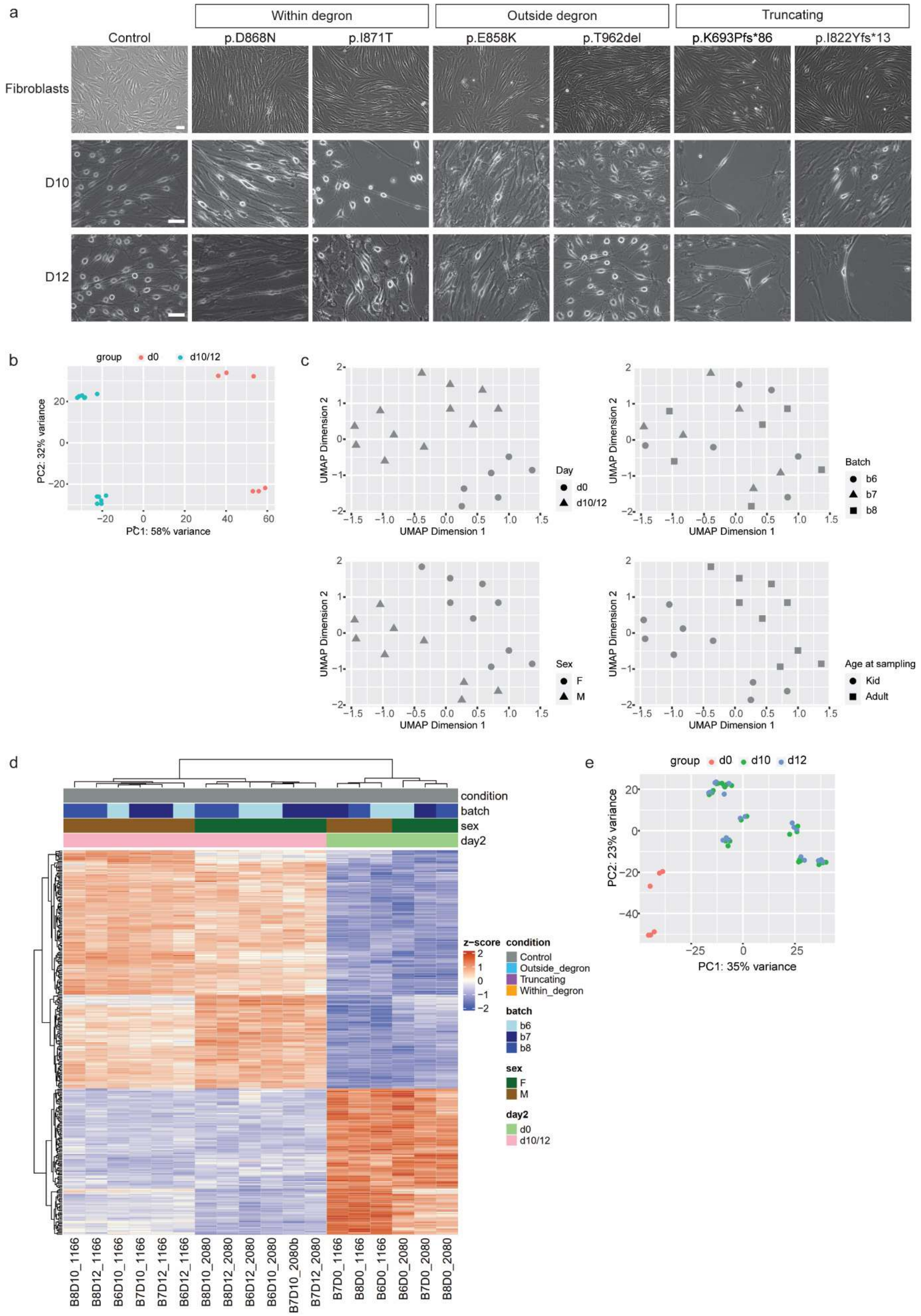

Supplementary Figure 11: Generation of induced neurons from fibroblasts.

a) Brightfield images of fibroblasts, D10 and D12 induced neurons. Scalebar = 100 μm.

b) Principal component analysis plot of fibroblasts and D10/12 induced neurons labelled by days *in vitro*.

c) UMAP plots of control fibroblasts and D10/12 induced neurons (labelled with days in vitro, batches, sex or age of sampling).

d) Heatmap of top 300 differentially expressed genes in D10/12 control induced neurons compared to control fibroblasts.

e) Principal component analysis plot of control and patient fibroblasts and D10/12 induced neurons labelled by days *in vitro*.

Source data are provided as a Source Data file.

Supplementary Figure 12

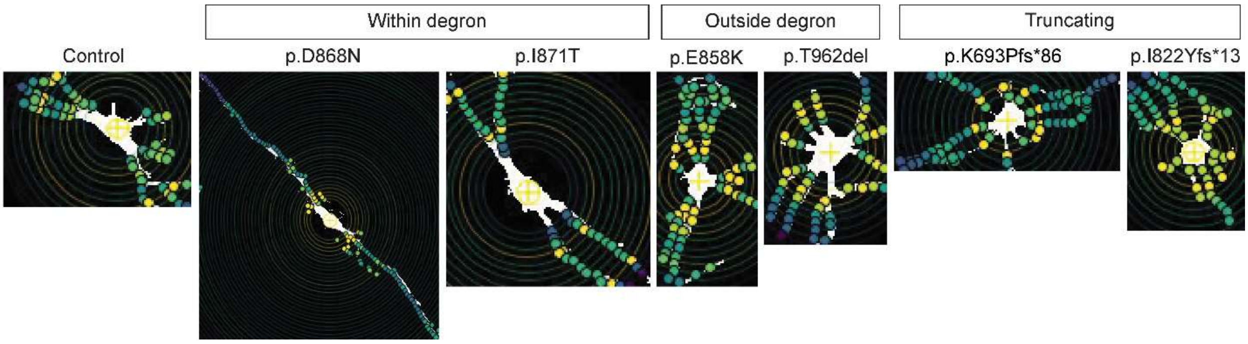

Supplementary Figure 12: Morphological analysis of day 12 induced neurons.
Representative somatodendritic reconstruction of control and induced neurons with *SETBP1* variants at D12 with 6-µm Sholl rings placed from the centre soma outward.
Source data are provided as a Source Data file.

Supplementary Figure 13

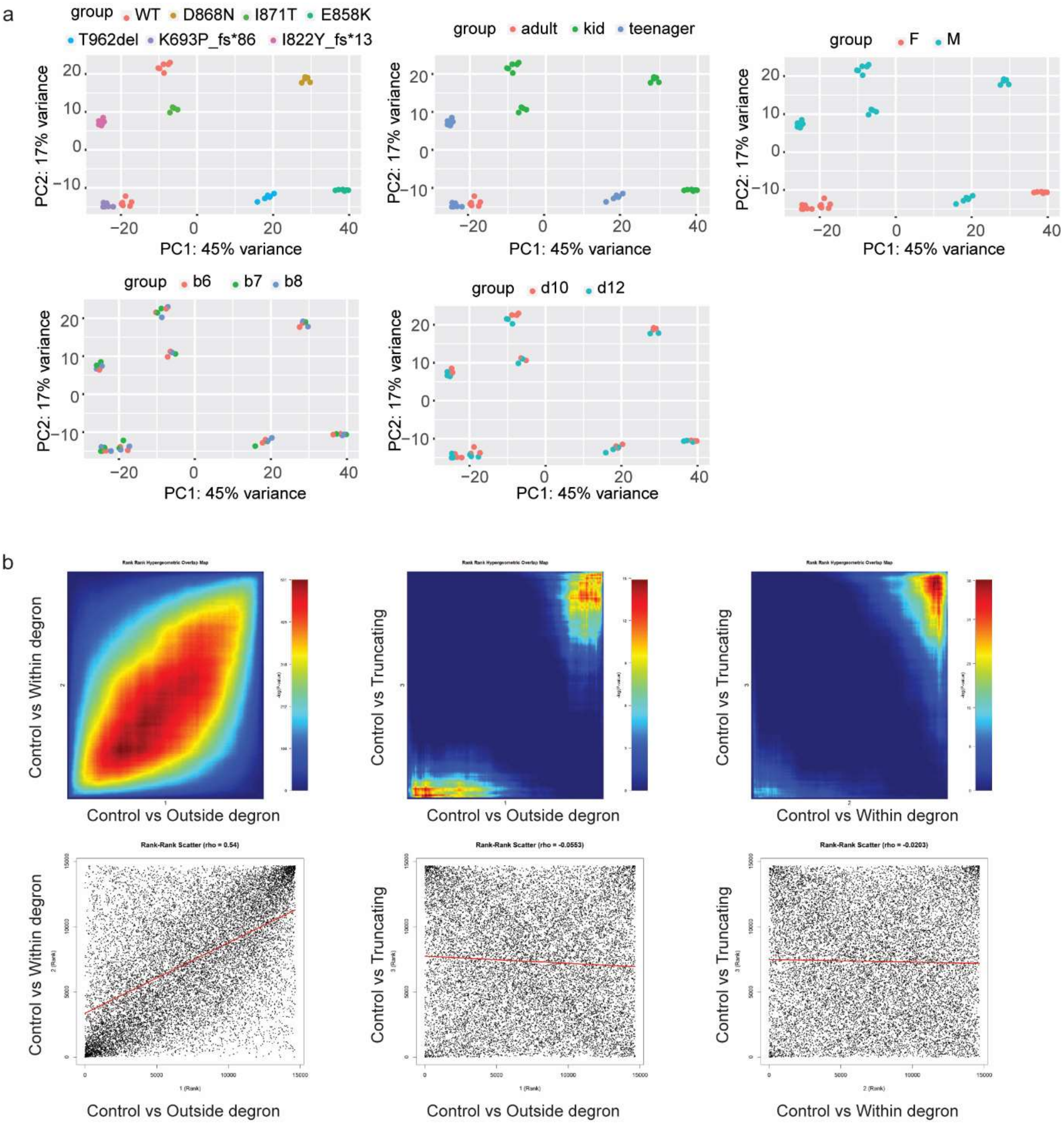

Supplementary Figure 13: RNA-seq analyses of induced neurons.

a) PCA plots of variance distribution of D10/12 induced neurons included in RNA-seq experiments. Three independent differentiation experiments were included.
Principal component (PC) 1 and PC2 account for 45% and
17% of total variance respectively. Samples were coloured by genotype, age group at sampling, biological sex, batch of differentiation experiments (b6-b8) and timepoints (d10 and d12) (left to right). A list of cell lines and samples included can be found in Supplementary Data 8. Kid = age 0-12; teenager = age 13-20; adult = age >20. F = female; M = male.

b) Heatmaps (top row) and scatter plots (bottom row) of RRHO analyses. From left to right, RRHO analyses showed moderate overlap between transcriptomic profiles of outside-degron and within-degron/SGS induced neurons (left), weak overlap in general but similar top up- and down-regulated genes between transcriptomic profiles of outside-degron and truncating induced neurons (middle), and weakest overlap in general but similar top down-regulated genes between transcriptomic profiles of within-degron/SGS and truncating induced neurons (right). *Rho* indicates Spearman rank correlation coefficient between two comparisons.

Source data are provided as a Source Data file.

Supplementary Figure 14

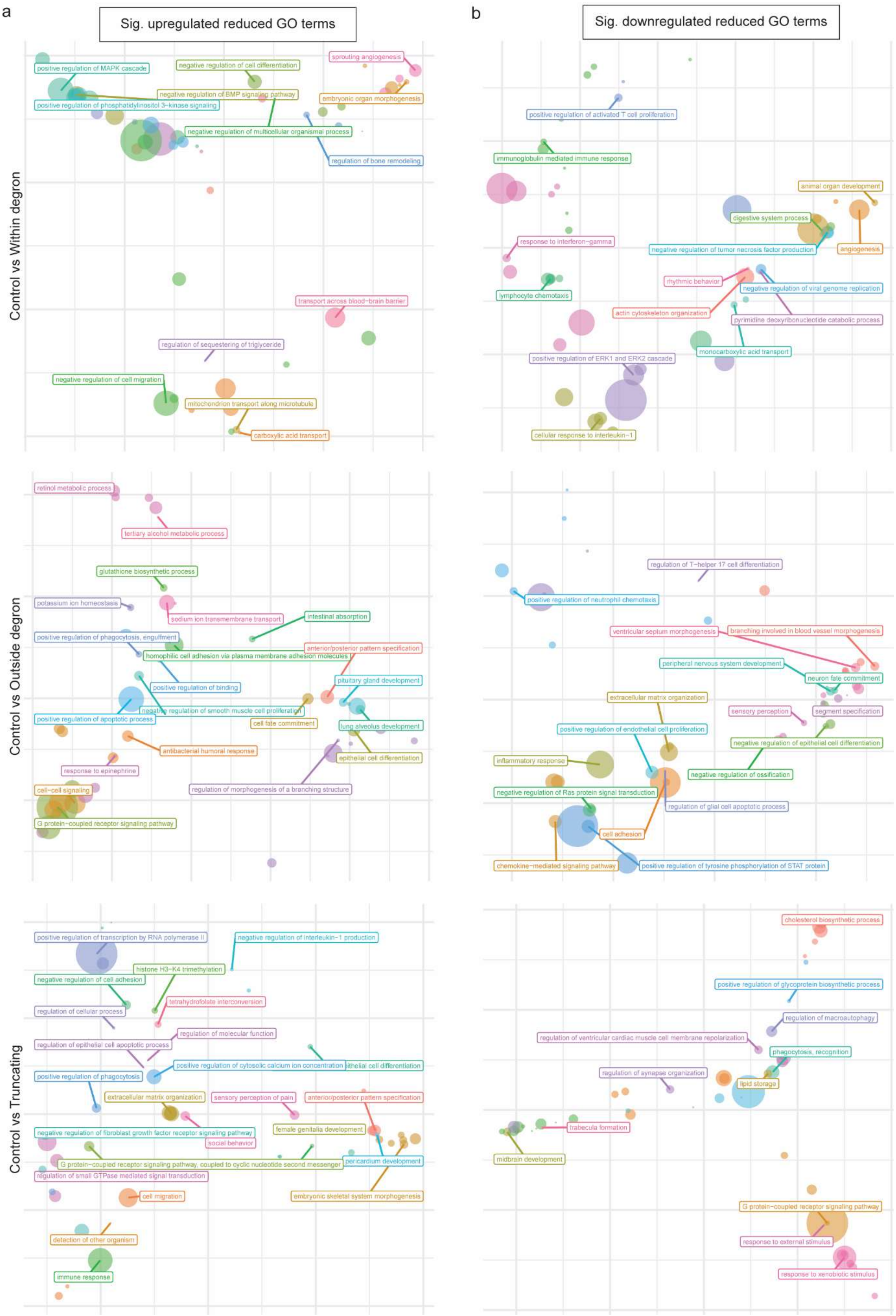

Supplementary Figure 14: Top 50 reduced gene ontology (GO) terms in biological pathways of significant differentially expressed genes of each variant group

a) Scatter plots of reduced GO terms in biological pathways of significantly up-regulated genes. b) Scatter plots of reduced GO terms in biological pathways of significantly down-regulated genes.

Source data are provided as a Source Data file.

Supplementary references

1. Silk, M., Petrovski, S. & Ascher, D. B. MTR-Viewer: identifying regions within genes under purifying selection. *Nucleic Acids Research* **47**, W121–W126 (2019).
2. Piazza, R. *et al.* SETBP1 induces transcription of a network of development genes by acting as an epigenetic hub. *Nat Commun* **9**, 2192 (2018).
3. Becker, M., Devanna, P., Fisher, S. E. & Vernes, S. C. Mapping of Human FOXP2 Enhancers Reveals Complex Regulation. *Frontiers in Molecular Neuroscience* **11**, 47 (2018).
4. Yang, Y. *et al.* Rapid and Efficient Conversion of Human Fibroblasts into Functional Neurons by Small Molecules. *Stem Cell Reports* **13**, 862–876 (2019).

Source data for Supplementary Fig 3D

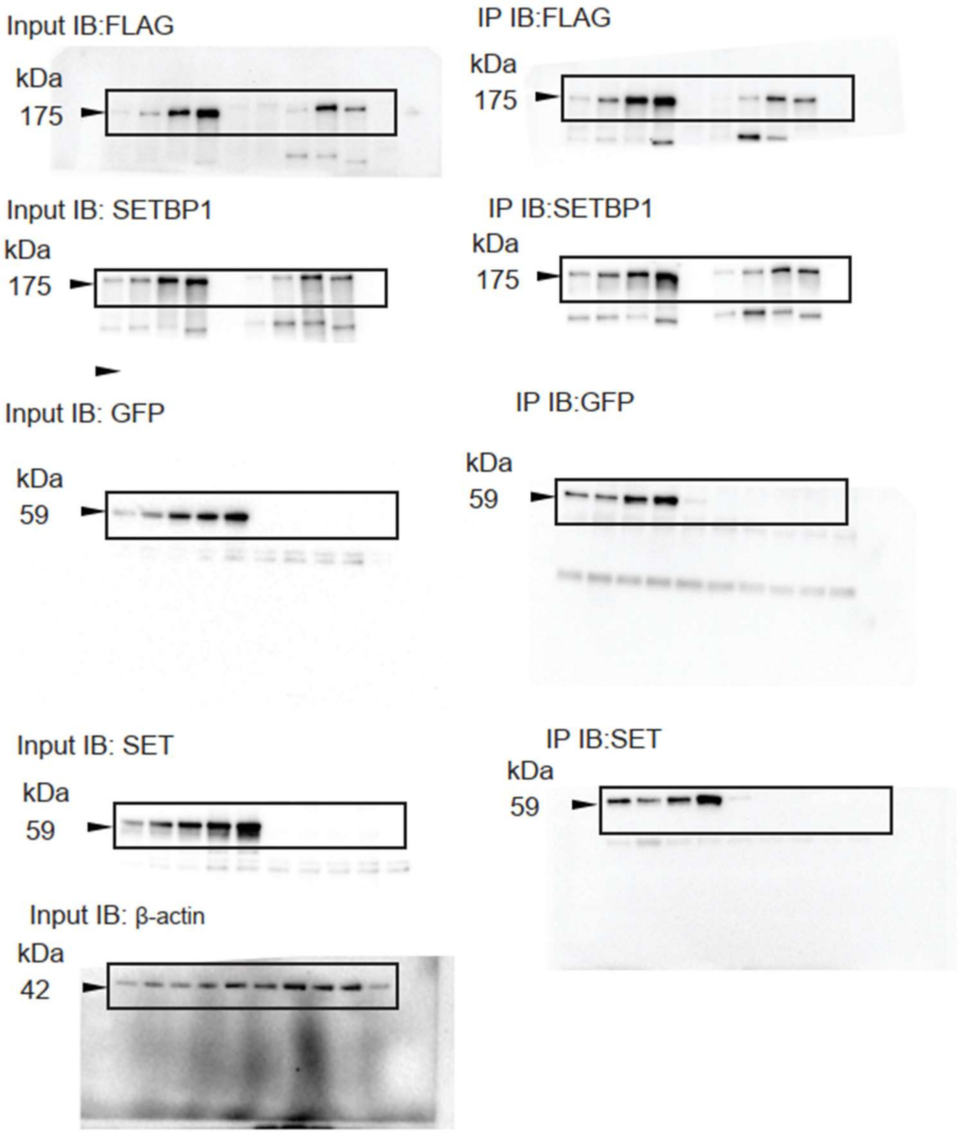

Supplement: Supplementary file 1 — Supplementary Information [file 41467_2025_64074_MOESM1_ESM.pdf]
