# Supplementary material for: SETBP1 variants outside the degron disrupt DNA-binding, transcription and neuronal differentiation capacity to cause a heterogeneous neurodevelopmental disorder
Source: Nat Commun. 2025 Oct 10;16:9021. doi: 10.1038/s41467-025-64074-x (PMC12514306; doi:10.1038/s41467-025-64074-x)
Supplement: Supplementary file 2 — Description of Additional Supplementary Files [file 41467_2025_64074_MOESM2_ESM.docx]

**Description of Additional Supplementary Files**

File Name: Supplementary Data 1; 41467_2025_64074_MOESM3_ESM.xlsx

Description: Clinical information per individual.

N/A: not assessed or not available; DD: developmental delay; ID: intellectual disability. Sequencing methods indicated are for reference only. Variants or patients previously reported are indicated^1–3^. Patients identified via diagnostic molecular analysis are indicated.

File Name: Supplementary Data 2; 41467_2025_64074_MOESM4_ESM.xlsx

Description: Summary of prediction of PhenoScore, HPO terms and facial features for individuals with *SETBP1* variants outside the degron.

SGS = 1, LoF = 0. If prediction score is higher 0.7, it is classified as similar to SGS; 0.3-0.7: No match; < 0.3: classified as similar to LoF; N/A: not applicable.

File Name: Supplementary Data 3; 41467_2025_64074_MOESM5_ESM.xlsx

Description: List of variants, including annotations and classification based on ACMG guidelines.

File Name: Supplementary Data 4; 41467_2025_64074_MOESM6_ESM.xlsx

Description: Frequency of variants in cancer.

File Name: Supplementary Data 5; 41467_2025_64074_MOESM7_ESM.xlsx

Description: Fibroblast cell lines included in RNA-seq.

Age at sampling: 1-12: kids; 13-20 teenagers; >20: adults. Related to Fig 5.

File Name: Supplementary Data 6; 41467_2025_64074_MOESM8_ESM.xlsx

Description: List of small molecules used for generating induced neurons from fibroblasts^4^.

File Name: Supplementary Data 7; 41467_2025_64074_MOESM9_ESM.xlsx

Description: Fibroblast cell lines included in generation of induced neurons using small molecules and included in RNA-seq.

Age at sampling: 1-12: kids; 13-20 teenagers; >20: adults. Related to Fig 6.

File Name: Supplementary Data 8; 41467_2025_64074_MOESM10_ESM.xlsx

Description: Sequences of primers and oligos used in the current study. F=forward primer; R=reverse primer; S=sense; AS=anti-sense; SDM=Site-Directed Mutagenesis.

File Name: Supplementary Data 9; 41467_2025_64074_MOESM11_ESM.xlsx

Description: List of antibodies used in the current study.^4^

Ms = mouse; Ck = chicken; Rb = rabbit; Gt = goat; Gp = guinea pig; IB = immunoblotting; IF = immunofluorescence

Reference

1. Hildebrand, M. S. *et al.* Severe childhood speech disorder: Gene discovery highlights transcriptional dysregulation. *Neurology* **94**, e2148–e2167 (2020).

2. Acuna-Hidalgo, R. *et al.* Overlapping SETBP1 gain-of-function mutations in Schinzel-Giedion syndrome and hematologic malignancies. *PLoS Genet.* **13**, e1006683 (2017).

3. Palmer, E. E. *et al.* Diagnostic Yield of Whole Genome Sequencing After Nondiagnostic Exome Sequencing or Gene Panel in Developmental and Epileptic Encephalopathies. *Neurology* **96**, e1770–e1782 (2021).

4. Yang, Y. *et al.* Rapid and Efficient Conversion of Human Fibroblasts into Functional Neurons by Small Molecules. *Stem Cell Rep.* **13**, 862–876 (2019).
