# Supplementary material for: SETBP1 variants outside the degron disrupt DNA-binding, transcription and neuronal differentiation capacity to cause a heterogeneous neurodevelopmental disorder
Source: Nat Commun. 2025 Oct 10;16:9021. doi: 10.1038/s41467-025-64074-x (PMC12514306; doi:10.1038/s41467-025-64074-x)
Supplement: Supplementary file 12 — Reporting Summary [file 41467_2025_64074_MOESM12_ESM.pdf]

nature portfolio

Corresponding author(s): Maggie MK Wong, Simon E FIsher

Last updated by author(s): Jul 9, 2025

Reporting Summary

Nature Portfolio wishes to improve the reproducibility of the work that we publish. This form provides structure for consistency and transparency in reporting. For further information on Nature Portfolio policies, see our Editorial Policies and the Editorial Policy Checklist.

Statistics

For all statistical analyses, confirm that the following items are present in the figure legend, table legend, main text, or Methods section.

n/a	Confirmed	
☐	☒	The exact sample size (*n*) for each experimental group/condition, given as a discrete number and unit of measurement
☐	☒	A statement on whether measurements were taken from distinct samples or whether the same sample was measured repeatedly
☐	☒	The statistical test(s) used AND whether they are one- or two-sided<br>*Only common tests should be described solely by name; describe more complex techniques in the Methods section.*
☒	☐	A description of all covariates tested
☒	☐	A description of any assumptions or corrections, such as tests of normality and adjustment for multiple comparisons
☐	☒	A full description of the statistical parameters including central tendency (e.g. means) or other basic estimates (e.g. regression coefficient) AND variation (e.g. standard deviation) or associated estimates of uncertainty (e.g. confidence intervals)
☐	☒	For null hypothesis testing, the test statistic (e.g. *F*, *t*, *r*) with confidence intervals, effect sizes, degrees of freedom and *P* value noted<br>*Give P values as exact values whenever suitable.*
☒	☐	For Bayesian analysis, information on the choice of priors and Markov chain Monte Carlo settings
☒	☐	For hierarchical and complex designs, identification of the appropriate level for tests and full reporting of outcomes
☒	☐	Estimates of effect sizes (e.g. Cohen's *d*, Pearson's *r*), indicating how they were calculated

Our web collection on statistics for biologists contains articles on many of the points above.

Software and code

Policy information about availability of computer code

Data collection	No software was used for data collection
Data analysis	All code, software and online resources used were stated in the Methods and Materials section.<br>Online tools:<br>CADD, https://cadd.gs.washington.edu<br>COSMIC, https://cancer.sanger.ac.uk/cosmic<br>DECIPHER, https://www.deciphergenomics.org/gene/SETBP1/<br>gnomAD, https://gnomad.broadinstitute.org<br>MetaDome, https://stuart.radboudumc.nl/metadome<br>MTR-Viewer, https://biosig.lab.uq.edu.au/mtr-viewer/<br>OMIM, https://www.omim.org<br>Spatial Clustering, https://github.com/laurensvdwiel/SpatialClustering<br>UniProt, https://www.uniprot.org<br>PhenoScore, https://github.com/ldingemans/PhenoScore<br><br>RNA-seq data analysis and visualisation:<br>https://github.com/galagoz/setbp1-missense.<br>R Statistical Software v4.0.3 and v4.1.2AnnotationDbi_1.56.2<br>AnnotationFilter_1.18.0<br>Biobase_2.54.0<br>BiocGenerics_0.40.0

biomaRt_2.50.3
circlize_0.4.16
ComplexHeatmap_2.10.0
cowplot_1.1.1
DESeq2_1.34.0
dplyr_1.1.2
EnhancedVolcano_1.12.0
EnsDb.Hsapiens.v79_2.99.0
ensembldb_2.18.4
forcats_1.0.0
futile.logger_1.4.3
GenomeInfoDb_1.30.1
GenomicFeatures_1.46.5
GenomicRanges_1.46.1
ggbeeswarm_0.7.2
ggplot2_3.3.6
ggrepel_0.9.3
GO.db_3.14.0
graph_1.72.0
gridExtra_2.3
hisat2_2.0.5
IRanges_2.28.0
lubridate_1.9.2
MatrixGenerics_1.6.0
matrixStats_0.63.0
org.Hs.eg.db_3.14.0
purrr_1.0.1
readr_2.1.4
RRHO_1.34.0
rrvgo_1.6.0
S4Vectors_0.32.4
SparseM_1.81
stringr_1.5.1
SummarizedExperiment_1.24.0
tibble_3.2.1
tidyr_1.3.0
tidyverse_2.0.0
topGO_2.46.0
umap_0.2.10.0
VennDiagram_1.7.3
vsn_3.62.0
biomaRt_2.46.3
Rsubread_2.4.3
PanelApp_intellectual_3.2

ImageJ/Fiji v.1.53e was used for Western blot quantitative analysis.
GraphPad Prism v8 was for statistical analysis of HEK cell-based experiments.

For manuscripts utilizing custom algorithms or software that are central to the research but not yet described in published literature, software must be made available to editors and reviewers. We strongly encourage code deposition in a community repository (e.g. GitHub). See the Nature Portfolio guidelines for submitting code & software for further information.

Data

Policy information about availability of data

All manuscripts must include a data availability statement. This statement should provide the following information, where applicable:

- Accession codes, unique identifiers, or web links for publicly available datasets
- A description of any restrictions on data availability
- For clinical datasets or third party data, please ensure that the statement adheres to our policy

The authors confirm that all the data supporting the findings in this study are available within the article and its supplementary material. The raw and intermediary RNA-seq data were deposited to the public data archive of the Max Planck Institute (Nijmegen), accession link: https://hdl.handle.net/1839/2bd2487c-ea79-4618-8245-5b15fae44970, as also described in the section “Data Availability” in our manuscript. Processed RNA-seq data are publicly available in the GEO database, as also described in the manuscript. The reference genome and the annotation file (ensembl_homo_sapiens_grch38_p12_gca_000001405_27) were downloaded from ensembl.

Research involving human participants, their data, or biological material

Policy information about studies with human participants or human data. See also policy information about sex, gender (identity/presentation), and sexual orientation and race, ethnicity and racism.

Reporting on sex and gender: Biological sex of patients are indicated. Since there is not biological sex bias in patients with SETBP1 variants, biological sex

Reporting on sex and gender	was included as a variable in differential gene expression analysis in RNA-seq analysis to control for potential effects on gene expression.
Reporting on race, ethnicity, or other socially relevant groupings	Patients with a molecular diagnosis of a SETBP1 variant, regardless of ethnicity, are included. This information could be used as an indirect identifier for an ultra rare patient population and is therefore not indicated in the Supplementary Data 1.
Population characteristics	Patients with a molecular diagnosis of a SETBP1 variant. Only patients with missense variants outside the degron region (not affecting amino acids 868-871) of SETBP1 were included in this study.
Recruitment	The clinical data and patient materials were obtained through international collaborations involving clinicians from various countries (Supplementary Table S1). Some of these collaborations were established via GeneMatcher. Given the rarity of the disease we do not perform statistical calculation on the patient population and therefore no biases (including self-selection bias) are applied here.
Ethics oversight	Written and informed consents were obtained from all patients or legal guardians. Consent was also obtained for publication of patient photos, where appropriate. Consent procedures were in accordance with the Declaration of Helsinki and local ethical guidelines of participating centres. The Human Research Ethics Committee of The Royal Children's Hospital, Melbourne, Australia, approved study and testing on a research basis of proband 1 (Project 37353). The other aspects of the study were under oversight and approval of the Medical Ethics Committee of the Radboud University Nijmegen Medical Center, Nijmegen, The Netherlands.

Note that full information on the approval of the study protocol must also be provided in the manuscript.

Field-specific reporting

Please select the one below that is the best fit for your research. If you are not sure, read the appropriate sections before making your selection.

☒ Life sciences ☐ Behavioural & social sciences ☐ Ecological, evolutionary & environmental sciences

For a reference copy of the document with all sections, see nature.com/documents/nr-reporting-summary-flat.pdf

Life sciences study design

All studies must disclose on these points even when the disclosure is negative.

Sample size	For all performed experiments we used equal to or greater than the generally accepted standard of three biological replications per group. All experiments performed in HEK cells and induced neuron differentiation were performed three times. For luciferase reporter assays, in each experiment for each condition, triplicates were included and each experiment was repeated three times. Three technical replicates of each fibroblast cell line were included in the RNA-seq experiments. A list of cell lines included can be found in Supplementary Data files. We specified the number of biological replicates and independent experiments in the respective figure legends.
Data exclusions	Outliers in RNAseq analyses of induced neurons were excluded. Apart from this, no data were excluded for other experiments or analyses.
Replication	We repeated all experiments using at least three biological replicates over independent experiments. The number of experimental replicates are listed and detailed in the methods and material section, and in figure legends when applicable. All attempts at replication were successful.
Randomization	We plated cells in a random distribution onto cell culture and multi-well plate positions, and randomly assigned them to experimental groups. Fibroblast samples collected from healthy donors were allocated as controls and those collected from patients were allocated in subgroups according to the variant types in molecular diagnosis, i.e. missense variants outside degron, missense variants within degron, or truncating variants. Biological sex, batch of sequencing and age group were included as variables when performing differential gene expression analysis to control for these effects. Untransfected control, empty vector control, and vehicle-only control were included in all cell-based experiments to control for possible background effects.
Blinding	The investigator who performed the RNA-seq data analysis was blinded to the genotype. Other data collection and analyses were collected in multi-well reader simultaneously. The same results have been repeated by multiple members of the research team.

Behavioural & social sciences study design

All studies must disclose on these points even when the disclosure is negative.

Study description	*Briefly describe the study type including whether data are quantitative, qualitative, or mixed-methods (e.g. qualitative cross-sectional, quantitative experimental, mixed-methods case study).*
Research sample	*State the research sample (e.g. Harvard university undergraduates, villagers in rural India) and provide relevant demographic information (e.g. age, sex) and indicate whether the sample is representative. Provide a rationale for the study sample chosen. For studies involving existing datasets, please describe the dataset and source.*
Sampling strategy	*Describe the sampling procedure (e.g. random, snowball, stratified, convenience). Describe the statistical methods that were used to predetermine sample size OR if no sample-size calculation was performed, describe how sample sizes were chosen and provide a*

	rationale for why these sample sizes are sufficient. For qualitative data, please indicate whether data saturation was considered, and what criteria were used to decide that no further sampling was needed.
Data collection	*Provide details about the data collection procedure, including the instruments or devices used to record the data (e.g. pen and paper, computer, eye tracker, video or audio equipment) whether anyone was present besides the participant(s) and the researcher, and whether the researcher was blind to experimental condition and/or the study hypothesis during data collection.*
Timing	*Indicate the start and stop dates of data collection. If there is a gap between collection periods, state the dates for each sample cohort.*
Data exclusions	*If no data were excluded from the analyses, state so OR if data were excluded, provide the exact number of exclusions and the rationale behind them, indicating whether exclusion criteria were pre-established.*
Non-participation	*State how many participants dropped out/declined participation and the reason(s) given OR provide response rate OR state that no participants dropped out/declined participation.*
Randomization	*If participants were not allocated into experimental groups, state so OR describe how participants were allocated to groups, and if allocation was not random, describe how covariates were controlled.*

Ecological, evolutionary & environmental sciences study design

All studies must disclose on these points even when the disclosure is negative.

Study description	*Briefly describe the study. For quantitative data include treatment factors and interactions, design structure (e.g. factorial, nested, hierarchical), nature and number of experimental units and replicates.*
Research sample	*Describe the research sample (e.g. a group of tagged Passer domesticus, all Stenocereus thurberi within Organ Pipe Cactus National Monument), and provide a rationale for the sample choice. When relevant, describe the organism taxa, source, sex, age range and any manipulations. State what population the sample is meant to represent when applicable. For studies involving existing datasets, describe the data and its source.*
Sampling strategy	*Note the sampling procedure. Describe the statistical methods that were used to predetermine sample size OR if no sample-size calculation was performed, describe how sample sizes were chosen and provide a rationale for why these sample sizes are sufficient.*
Data collection	*Describe the data collection procedure, including who recorded the data and how.*
Timing and spatial scale	*Indicate the start and stop dates of data collection, noting the frequency and periodicity of sampling and providing a rationale for these choices. If there is a gap between collection periods, state the dates for each sample cohort. Specify the spatial scale from which the data are taken*
Data exclusions	*If no data were excluded from the analyses, state so OR if data were excluded, describe the exclusions and the rationale behind them, indicating whether exclusion criteria were pre-established.*
Reproducibility	*Describe the measures taken to verify the reproducibility of experimental findings. For each experiment, note whether any attempts to repeat the experiment failed OR state that all attempts to repeat the experiment were successful.*
Randomization	*Describe how samples/organisms/participants were allocated into groups. If allocation was not random, describe how covariates were controlled. If this is not relevant to your study, explain why.*
Blinding	*Describe the extent of blinding used during data acquisition and analysis. If blinding was not possible, describe why OR explain why blinding was not relevant to your study.*

Did the study involve field work? ☐ Yes ☐ No

Field work, collection and transport

Field conditions	*Describe the study conditions for field work, providing relevant parameters (e.g. temperature, rainfall).*
Location	*State the location of the sampling or experiment, providing relevant parameters (e.g. latitude and longitude, elevation, water depth).*
Access & import/export	*Describe the efforts you have made to access habitats and to collect and import/export your samples in a responsible manner and in compliance with local, national and international laws, noting any permits that were obtained (give the name of the issuing authority, the date of issue, and any identifying information).*
Disturbance	*Describe any disturbance caused by the study and how it was minimized.*

Reporting for specific materials, systems and methods

We require information from authors about some types of materials, experimental systems and methods used in many studies. Here, indicate whether each material, system or method listed is relevant to your study. If you are not sure if a list item applies to your research, read the appropriate section before selecting a response.

Materials & experimental systems		
n/a	Involved in the study	
☐	☒	Antibodies
☐	☒	Eukaryotic cell lines
☐	☐	Palaeontology and archaeology
☐	☐	Animals and other organisms
☐	☒	Clinical data
☐	☐	Dual use research of concern
☐	☐	Plants

Methods		
n/a	Involved in the study	
☐	☐	ChIP-seq
☐	☐	Flow cytometry
☐	☐	MRI-based neuroimaging

Antibodies

Antibodies used

A list of antibodies and dilution used in this study is provided in Supplementary Table 8. β-actin Ms Sigma-Aldrich A5441; DKK/FLAG Ms Origene TA50011-100; JL-8 Ms Clonetech 632380; Ki-67 Ck EnCor Biotech CPCA-Ki67; phosphorylated PP2A (Tyr307, clone E155) Rb abcam ab32104; PP2A Antibody, C subunit, clone 1D6 Ms Millipore 05-421; SET Rb Abcam ab1183; SETBP1 Rb ProteinTech 16841-1-AP; SETBP1 Rb Atlas Antibodies HPA049022; Ubiquitin Rb Cell Signalling 43124S; AlexaFluor 488-conjugated-anti-rabbit IgG Invitrogen A11034; AlexaFluor 568-conjugated-anti-mouse IgG Invitrogen A11031; AlexaFluor 568-conjugated-anti-rabbit IgG anti-rabbit IgG Gt Invitrogen A11036; AlexaFluor 647-conjugated-anti-chicken IgG Invitrogen A21449; HRP-conjugated-anti-rabbit IgG Jackson ImmunoResearch 111-035-003; HRP-conjugated-anti-mouse IgG Jackson ImmunoResearch 115-007-003; Tuj1 Gp Synaptic Systems 302 304; MAP2 Ck Abcam Ab5392; DCX Rb Abcam ab18723; Alexa Fluor 488-conjugated-anti- Guinea Pig IgG Gt Invitrogen A11073

Validation

All antibodies used in this work are commercial and tested in literature for the proposed assay.
β-actin Sigma-Aldrich A5441
https://www.sigmaaldrich.com/NL/en/product/sigma/a5441

DKK/FLAG Origene TA50011-100
https://www.origene.com/catalog/antibodies/tag-antibodies/ta50011-100/clone-oti4c5-anti-ddk-flag-monoclonal-antibody

JL-8 Clonetech 632380
https://www.takarabio.com/documents/Certificate%20of%20Analysis/632380/632380-632381-070313.pdf

Ki-67 EnCor Biotech CPCA-Ki67
https://encorbio.com/product/cpca-ki67/

phosphorylated PP2A (Tyr307, clone E155) abcam ab32104
https://www.abcam.com/pp2a-alpha--beta-antibody-e155-ab32104.html

PP2A Antibody, C subunit, clone 1D6 Millipore 05-421
https://www.merckmillipore.com/NL/en/product/Anti-PP2A-Antibody-C-subunit-clone-1D6,MM_NF-05-421?ReferrerURL=https%3A%2F%2Fwww.google.com%2F

SET Abcam ab1183
https://www.abcam.com/settaf-i-antibody-ab1183.html

SETBP1 ProteinTech 16841-1-AP;
https://www.ptglab.com/products/SETBP1-Antibody-16841-1-AP.htm

SETBP1 Atlas Antibodies HPA049022
https://www.atlasantibodies.com/products/antibodies/primary-antibodies/triple-a-polyclonals/setbp1-antibody-hpa049022/

Ubiquitin Cell Signalling 43124S
https://www.cellsignal.com/products/primary-antibodies/ubiquitin-e4i2j-rabbit-mab/43124

AlexaFluor 488-conjugated-anti-rabbit IgG Invitrogen A11034
https://www.thermofisher.com/antibody/product/Goat-anti-Rabbit-IgG-H-L-Highly-Cross-Adsorbed-Secondary-Antibody-Polyclonal/A-11034

AlexaFluor 568-conjugated-anti-mouse IgG Invitrogen A11031
https://www.thermofisher.com/antibody/product/Goat-anti-Mouse-IgG-H-L-Highly-Cross-Adsorbed-Secondary-Antibody-Polyclonal/A-11031

AlexaFluor 568-conjugated-anti-rabbit IgG anti-rabbit IgG Gt Invitrogen A11036
https://www.thermofisher.com/antibody/product/Goat-anti-Rabbit-IgG-H-L-Highly-Cross-Adsorbed-Secondary-Antibody-Polyclonal/A-11036

AlexaFluor 647-conjugated-anti-chicken IgG Invitrogen A21449
https://www.thermofisher.com/antibody/product/Goat-anti-Chicken-IgY-H-L-Secondary-Antibody-Polyclonal/A-21449

HRP-conjugated-anti-rabbit IgG Jackson ImmunoResearch 111-035-003
https://www.jacksonimmuno.com/catalog/products/111-035-003

HRP-conjugated-anti-mouse IgG Jackson ImmunoResearch 115-007-003
https://www.jacksonimmuno.com/catalog/products/115-007-003

Eukaryotic cell lines

Policy information about cell lines and Sex and Gender in Research

Cell line source(s)	HEK293T/17 cells (CRL-11268, ATCC).<br>Patient and control fibroblasts were obtained from collaborators according to local ethics guidelines. SGS fibroblasts were derived in Acuna-Hidalgo, R. et al. Overlapping SETBP1 gain-of-function mutations in Schinzel-Giedion syndrome and hematologic malignancies. PLoS Genet. 13, e1006683 (2017). LoF patient fibroblasts were collected according to local ethics approval (Medical Ethics Committee of the Radboud University Nijmegen Medical Center, Nijmegen, The Netherlands).<br>GM27453 (purchased from NIGMS Human Genetic Cell Repository, Coriell Institute)<br>A list of human primary fibroblast lines used are included in Supplementary Data 5, age at sampling and sex is indicated.
Authentication	Fibroblasts were tested for mycoplasma and the presence of expected variants (Sanger sequencing) before their usage in experiments.
Mycoplasma contamination	All cell lines tested negative for mycoplasma (PCR-test).
Commonly misidentified lines<br>(See ICLAC register)	No commonly misidentified cell lines were used.

Palaeontology and Archaeology

Specimen provenance	Not applicable
Specimen deposition	Not applicable
Dating methods	Not applicable

☐ Tick this box to confirm that the raw and calibrated dates are available in the paper or in Supplementary Information.

Ethics oversight	Not applicable

Note that full information on the approval of the study protocol must also be provided in the manuscript.

Animals and other research organisms

Policy information about studies involving animals; ARRIVE guidelines recommended for reporting animal research, and Sex and Gender in Research

Laboratory animals	Not applicable
Wild animals	Not applicable
Reporting on sex	Not applicable
Field-collected samples	Not applicable
Ethics oversight	Not applicable

Note that full information on the approval of the study protocol must also be provided in the manuscript.

Clinical data

Policy information about clinical studies

All manuscripts should comply with the ICMJE guidelines for publication of clinical research and a completed CONSORT checklist must be included with all submissions.

Clinical trial registration	Not applicable
Study protocol	Not applicable
Data collection	Not applicable
Outcomes	Not applicable

Dual use research of concern

Policy information about dual use research of concern

Hazards

Could the accidental, deliberate or reckless misuse of agents or technologies generated in the work, or the application of information presented in the manuscript, pose a threat to:

No	Yes	
☒	☐	Public health
☒	☐	National security
☒	☐	Crops and/or livestock
☒	☐	Ecosystems
☒	☐	Any other significant area

Experiments of concern

Does the work involve any of these experiments of concern:

No	Yes	
☒	☐	Demonstrate how to render a vaccine ineffective
☒	☐	Confer resistance to therapeutically useful antibiotics or antiviral agents
☒	☐	Enhance the virulence of a pathogen or render a nonpathogen virulent
☒	☐	Increase transmissibility of a pathogen
☒	☐	Alter the host range of a pathogen
☒	☐	Enable evasion of diagnostic/detection modalities
☒	☐	Enable the weaponization of a biological agent or toxin
☒	☐	Any other potentially harmful combination of experiments and agents

Plants

Seed stocks	Not applicable
Novel plant genotypes	Not applicable
Authentication	Not applicable

ChIP-seq

Data deposition

☐ Confirm that both raw and final processed data have been deposited in a public database such as GEO.

☐ Confirm that you have deposited or provided access to graph files (e.g. BED files) for the called peaks.

Data access links<br>*May remain private before publication.*	Not applicable
Files in database submission	Not applicable
Genome browser session<br>(e.g. UCSC)	Not applicable

Methodology

Replicates	Not applicable
Sequencing depth	Not applicable
Antibodies	Not applicable
Peak calling parameters	Not applicable
Data quality	Not applicable
Software	Not applicable

Flow Cytometry

Plots

Confirm that:

☐ The axis labels state the marker and fluorochrome used (e.g. CD4-FITC).

☐ The axis scales are clearly visible. Include numbers along axes only for bottom left plot of group (a 'group' is an analysis of identical markers).

☐ All plots are contour plots with outliers or pseudocolor plots.

☐ A numerical value for number of cells or percentage (with statistics) is provided.

Methodology

Sample preparation	Not applicable
Instrument	Not applicable
Software	Not applicable
Cell population abundance	Not applicable
Gating strategy	Not applicable

☐ Tick this box to confirm that a figure exemplifying the gating strategy is provided in the Supplementary Information.

Magnetic resonance imaging

Experimental design

Design type	Not applicable
Design specifications	Not applicable
Behavioral performance measures	Not applicable

Acquisition

Imaging type(s)	Not applicable
Field strength	Not applicable
Sequence & imaging parameters	Not applicable
Area of acquisition	Not applicable

Diffusion MRI ☐ Used ☐ Not used

Preprocessing

Preprocessing software	Not applicable
Normalization	Not applicable
Normalization template	Not applicable
Noise and artifact removal	Not applicable
Volume censoring	Not applicable

Statistical modeling & inference

Model type and settings	Not applicable
Effect(s) tested	Not applicable

Specify type of analysis: ☐ Whole brain ☐ ROI-based ☐ Both

Statistic type for inference (See Eklund et al. 2016)	Not applicable
Correction	Not applicable

Models & analysis

n/a	Involved in the study
☐	☐ Functional and/or effective connectivity
☐	☐ Graph analysis
☐	☐ Multivariate modeling or predictive analysis

Functional and/or effective connectivity	Not applicable
Graph analysis	Not applicable
Multivariate modeling and predictive analysis	Not applicable
