# Supplementary material for: SETBP1 variants outside the degron disrupt DNA-binding, transcription and neuronal differentiation capacity to cause a heterogeneous neurodevelopmental disorder
Source: Nat Commun. 2025 Oct 10;16:9021. doi: 10.1038/s41467-025-64074-x (PMC12514306; doi:10.1038/s41467-025-64074-x)
Supplement: Supplementary file 14 — Source Data [file 41467_2025_64074_MOESM14_ESM.zip › SourceData/Source data front page.docx]

**Source Data**

This zip file includes:

File name: Source data front page

Description: A list of source data files included with a short description.

File name: Source Data 1

Description: Source data for spatial clustering analysis of *SETBP1* variants outside the degron

File name: Source Data 2

Description: Source data for Fig 1e

File name: Source Data 3

Description: Source data for Fig 2

File name: Source Data 4

Description: Source data for Fig 3 and Supp Fig 5-7

File name: Source Data 5

Description: Source data for Fig 4 and Supp Fig 8

File name: Source Data 6

Description: Source data for Fig 5c: Significant differentially expressed genes in fibroblasts carrying *SETBP1* variants within the degron/SGS, outside degron and truncating variants compared to healthy controls (adjusted *p-value* <0.1 after multiple testing correction with FDR/Benjamini-Hochberg method, log_2_ fold change ≤ -1 or ≥ 1).

File name: Source Data 7

Description: Source data for Fig 5d: Lists of overlapping and total unique differentially expressed genes in fibroblasts carrying *SETBP1* variants within the degron/SGS, outside the degron, and truncating variants compared to healthy controls. Differentially expressed genes with an Ensembl ID but not a gene symbol in the Genome wide annotation for Human database^4^ were removed.

File name: Source Data 8

Description: Source data for Fig 5e: List of top 10 significantly upregulated and downregulated GO-terms in fibroblasts carrying *SETBP1* variants within the degron/SGS, outside the degron, and truncating variants compared to healthy controls. R package topGO^5^ was used (at least 10 genes in each GO-term, adjusted *p-value* <0.1, Fisher’s exact test with multiple testing correction). BP=biological processes; MF=molecular functions and CC=cellular components. Gene ontology categories with Benjamini-Hochberg FDR ≤ 0.05 using REVIGO^6^ are also shown.

File name: Source Data 9

Description: Source data for Fig 6f-g: Cell counting of days 10 and 12 neurons and morphological analysis of day 12 neurons (adjusted *p-value* <0.1 after multiple testing correction with FDR/Benjamini-Hochberg method, log_2_ fold change ≤ -1 or ≥ 1).

File name: Source Data 10

Description: Source data for Fig 7b: Significant differentially expressed genes in day 10/12 induced neurons carrying *SETBP1* variants within the degron/SGS, outside the degron and truncating variants compared to healthy controls (adjusted *p-value* <0.1 after multiple testing correction with FDR/Benjamini-Hochberg method, log_2_ fold change ≤ -1 or ≥ 1).

File name: Source Data 11

Description: Source data for Fig 7d: List of top 20 significantly upregulated and downregulated GO-terms in day 10/12 induced neurons carrying *SETBP1* variants within the degron/SGS, outside the degron, and truncating variants compared to healthy controls. R package topGO^5^ was used (at least 10 genes in each GO-term, adjusted *p-value* <0.1, Fisher’s exact test with multiple testing correction). BP=biological processes; MF=molecular functions and CC=cellular components. Gene ontology categories with Benjamini-Hochberg FDR ≤ 0.05 using rrvgo^6^ are also shown.

File name: Source Data 12

Description: Source data for Supp Fig 2d. Input for calculating MTR score.

File name: Source Data 13

Description: Source data for Supp Fig 4

File name: Source Data 14

Description: Source data for Supp Fig 11: Significant differentially expressed genes in day 10/12 control induced neurons compared day 0 control fibroblasts (adjusted p-value <0.1 after multiple testing correction with FDR/Benjamini-Hochberg method, log2 fold change ≤ -1 or ≥ 1).

File name: Source Data 15

Description: Source data for enrichment analysis: Enrichment analysis of differentially expressed genes (within, outside degron, and truncating) in day 10/12 induced neurons against genes associated with autism (v.0.22) and intellectual disability (intellectual disability v3.2) from PanelApp database. Fisher-exact test was used to calculate enrichment, a *p*-value <0.05 is considered significant.

File name: Source Data 16

Description: Uncropped immunoblots for Fig 2-3.
