# Supplementary figures and images for: SETBP1 variants outside the degron disrupt DNA-binding, transcription and neuronal differentiation capacity to cause a heterogeneous neurodevelopmental disorder
Source: Nat Commun. 2025 Oct 10;16:9021. doi: 10.1038/s41467-025-64074-x (PMC12514306; doi:10.1038/s41467-025-64074-x)

**Source data for Fig 2**

**
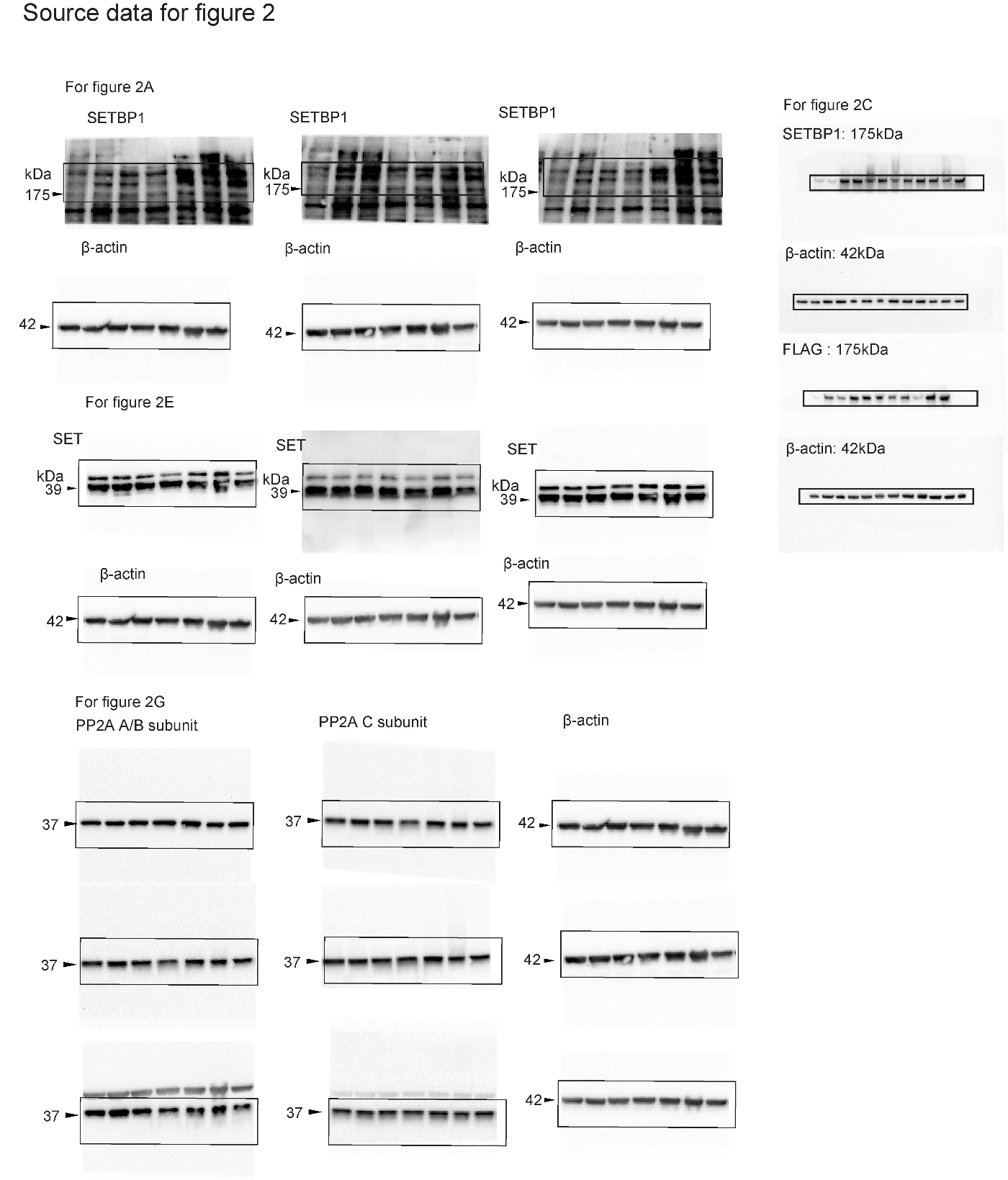
**

**Source data for Fig 3**

**
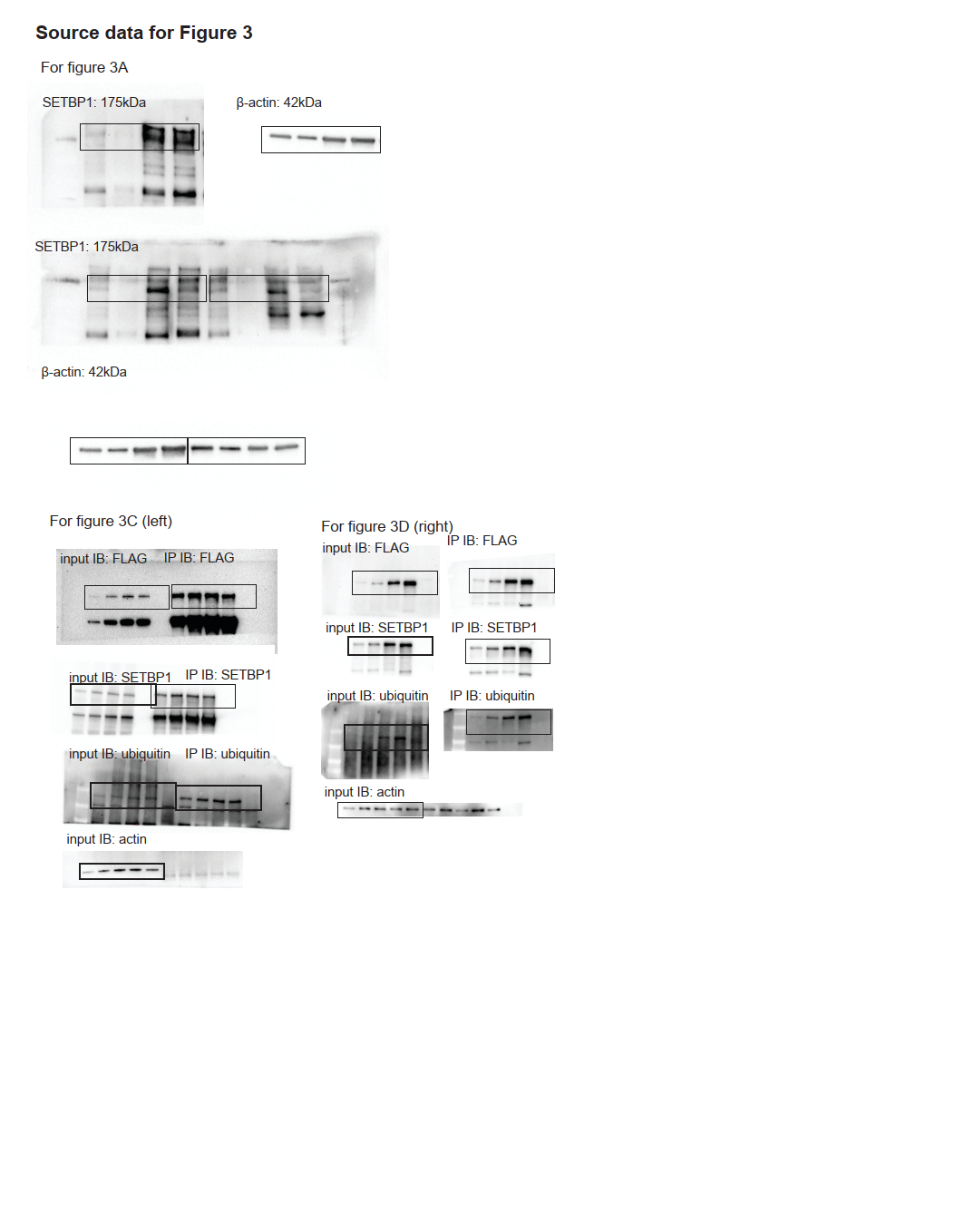
**

Supplement: Supplementary file 14 — Source Data [file 41467_2025_64074_MOESM14_ESM.zip › SourceData/SourceData16_forFig 2-3.docx]
